# Promoting Social Identities as Resources—The Role of Ethnic and National Identity Development for Adolescents' Global Identity Coherence in Ethnic‐Culturally Diverse Schools in Germany

**DOI:** 10.1002/jcop.70059

**Published:** 2025-11-14

**Authors:** Sharleen Pevec‐Zimmer, Tuğçe Aral, Linda P. Juang, Maja K. Schachner

**Affiliations:** ^1^ Department of Inclusive Education University of Potsdam Potsdam Germany; ^2^ Department of Educational Psychology Martin‐Luther‐University Halle‐Wittenberg Halle Germany

**Keywords:** cultural diversity, equity education, ethnic and national identities, ethnic minorities, identity coherence, longitudinal studies

## Abstract

Ethnic and national identities can be meaningful social identities promoting students' well‐being and academic engagement. Unclear is whether and how each identity contributes to ethnic‐culturally minoritized versus majoritized adolescents' global identity coherence. Using longitudinal data collected in three cohorts during 2018‐2022, we conducted multigroup mediation analyses, testing whether a school‐based identity intervention promoted ethnic and national identities (exploration, resolution) 1 week post‐intervention (T2), which in turn, promoted identity coherence (more synthesis, less confusion) 8 weeks postintervention (T3). The results showed that the intervention promoted ethnic identity exploration for both groups and ethnic identity resolution for majoritized adolescents. For both groups, ethnic identity resolution predicted less identity confusion. Only for majoritized adolescents, the intervention promoted ethnic identity resolution, which in turn promoted less identity confusion and more synthesis, perhaps due to ceiling effects of minoritized adolescents' ethnic identity resolution. Findings emphasize the role of students' ethnic identity for global identity coherence as a potential resource for their development. The role of national identity on global identity coherence is less clear.

For youth, developing a coherent identity and understanding who they are is important, especially during adolescence (Erikson 1968) and thus should be encouraged in school (Verhoeven et al. 2019). To develop a coherent sense of self, adolescents need to integrate and manage different social identities into an overall identity (Branje et al. 2021). In increasingly diverse societies that are shaped through migration, two potentially important social identities are based on adolescents' ethnicities (family heritages) and national belonging (Knight et al. 2018; Mastrotheodoros et al. 2021). Importantly, whether adolescents are part of ethnic‐culturally minoritized groups or the majoritized group^1^ can impact their ethnic and national identities and how these identities are integrated into an overall identity (Verkuyten 2009). Potential differences between minoritized versus majoritized adolescents include the salience of ethnic and national identities (Froehlich et al. 2020; Umaña‐Taylor et al. 2014; Verkuyten 2009), whether their ethnic and national identities are conflicting or compatible, distinct or overlapping (Martiny et al. 2017; Thijs et al. 2025), and whether a national identity group is inclusive or restricted (Ditlmann and Kopf‐Beck 2019; Moffitt et al. 2018).

Both social identities can be meaningful for minoritized and majoritized adolescents' overall identity and potentially contribute to their global identity coherence, which has not yet been extensively examined (Crocetti et al. 2018; Schwartz et al. 2013). It is important to do so as the positive effects of ethnic, and potentially national, identities on outcomes such as academic achievement and psychological well‐being may go through global identity coherence (Safa et al. 2024; Umaña‐Taylor et al. 2018). Thus, in this study, we examine how ethnic and national identity, fostered through opportunities for exploration and resolution in the school‐based Identity Project intervention (Umaña‐Taylor and Douglass 2017) in Germany, may contribute to minoritized and majoritized students' global identity coherence in terms of more synthesis and less confusion. We focus on how supporting ethnic and national identities potentially contribute to educational equity by addressing issues of belonging, inclusion and exclusion for minoritized and majoritized adolescents growing up in Germany (Alim et al. 2020). As research on ethnic(‐racial) identity^2^ development has predominantly been conducted in the United States especially since the 1990s (Rivas‐Drake, Seaton, et al. 2014), and the Identity Project was developed in the United States, we highlight key findings in the United States, European and Identity Project intervention studies and detail the sociocultural context in Germany.

## 1 Social Identities and Adolescents' Development of a Coherent Identity

Social identities, such as ethnic and national identities can be a source for adolescents' sense of self and provide guidance, for example, by offering shared traditions, values, knowledge, and history, which are communicated in interactions with others (Turner et al. 1987). Furthermore, how individuals group themselves and others, build categorizations and identify with social groups, is important for self development (Turner et al. 1987). It is assumed that individuals aim to maintain a positive evaluation of the social groups they belong to (Tajfel and Turner 1979). Negative perceptions of ingroups can provoke adolescents to maintain positive understandings of themselves and their social groups, for example, through rejecting or distancing themselves from negatively perceived groups or devaluing outgroups.

Hence, adolescents' sense of self can be impacted when they are confronted with members of and messages about social groups they feel they belong to, and members of social groups they feel they do not belong to, through exploring, committing, and reconsidering identities and the meaning for their own sense of self (Crocetti et al. 2018). Exploration captures actively thinking about and reflecting on identity domains as well as talking with others and seeking information. Commitment refers to making choices concerning identity domains and how these identity domains impact an individuals' self‐confidence. Reconsideration captures comparing current with alternative commitments when previous commitments are no longer satisfactory (Crocetti 2017). Coming to clarity, also referred to as resolution, coherence or cohesion, and making decisions by rejecting or adopting an identity domain is positively related to adolescents' well‐being (Branje 2022; Crocetti 2017). In addition, identity confusion and the reconsiderations of identity domains may be necessary for identity clarity in the longer term (Branje et al. 2021; De Lise et al. 2024). In immigration societies, two important social identity domains that may contribute to greater global identity coherence in terms of greater synthesis and less confusion, are ethnic and national identities.

## 2 Ethnic and National Identity as Resources for Minoritized and Majoritized Adolescents in School

In the United States, studies on ethnic(‐racial) and national identity investigate two broad groups: those who are often categorized and racialized as white, connected to societal privileges, and those who belong to an ethnic‐racial minoritized group with societal disadvantages. In contrast, European studies often group participants regarding their belonging or not belonging to the ethnic‐culturally constructed norm. This categorization is oftentimes based on the experience of immigration and family heritage cultures other than the country of residence, for example, nonimmigrant versus immigrant adolescents, adolescents without versus with a migration background, ethnic‐culturally majority versus ethnic‐culturally minoritized or marginalized adolescents. Although less commonly used, other categorizations can capture perceived ethnic‐racial discrimination, or open‐ended questions to assess participants' ethnic‐cultural self‐identifications (Vietze et al. 2023). Categorizations in ethnic(‐racial) identity research do not allow for individual or subgroup‐specific differentiations, yet they highlight societal (dis)advantages and (in)equities that people can experience *because* they belong to the majoritized ethnic‐cultural group or minoritized groups, and can affect the salience of and engagement with these groups.

### 2.1 Ethnic Identity

When studying ethnic(‐racial) identity development, different components reflect the processes by which it develops (Umaña‐Taylor et al. 2014). Exploration refers to students' information seeking about their ethnic(‐racial) group and engaging in activities and conversations to find out more about this certain social group membership. Resolution refers to students' coming to more clarity, after a period of exploration, about what their ethnic(‐racial) group belonging means to them. Consequently, both exploration and resolution are relevant for youth to come to an achieved and developed ethnic identity (Erikson 1968).

The benefits of adolescents engaging with their ethnic(‐racial) identities are seen through positive links with self‐esteem, life‐satisfaction, fewer depressive symptoms (Umaña‐Taylor et al. 2018) and academic engagement, achievement, and motivation (Miller‐Cotto and Byrnes 2016; Umaña‐Taylor et al. 2018). How adolescents can profit from their ethnic(‐racial) identity can differ depending on the racialized group and the ethnic(‐racial) identity component. For example, a meta‐analysis by Rivas‐Drake, Syed, et al. (2014) indicates that ethnic(‐racial) identity is positively associated with African American adolescents' psychological well‐being, academic adaptation and mental health, but shows that results are more mixed for other ethnic‐racial groups. Furthermore, the review highlights that not all components of ethnic(‐racial) identity are equally beneficial for adolescents' psychological, academic and health outcomes (Rivas‐Drake, Syed, et al. 2014). Yip et al. (2019), for example, concluded that adolescents' clarity about and positive evaluation of belonging to their ethnic(‐racial) group, but not exploration, can buffer against negative effects of ethnic‐racial discrimination. Overall, it is assumed that an achieved ethnic(‐racial) identity, where adolescents gain more clarity about their ethnic(‐racial) identities after learning more about it, is especially favorable (Umaña‐Taylor et al. 2018).

Interestingly, studies conducted in Germany replicate associations of minoritized adolescents' stronger ethnic identifications^3^ with higher life satisfaction and well‐being and fewer depressive symptoms, but do not find similar positive effects of ethnic identification on academic outcomes (Baumert et al. 2024; Hannover et al. 2013; Schotte et al. 2018). This may reflect the ongoing pressure of assimilationist approaches in educational contexts in Germany, where minoritized students' may reject their family heritage cultures in favor of the national German identification as a strategy for adaptation (Hannover et al. 2013). Overall, however, minoritized adolescents' ethnic identities can help develop resilience in societal contexts where they confront racialized experiences, marginalization and ethnic‐racial discrimination (Hoffman and Umaña‐Taylor 2023; Rivas‐Drake et al. 2022; Umaña‐Taylor et al. 2014; Umaña‐Taylor and Rivas‐Drake 2021).

As ethnic(‐racial) identities are considered more salient for minoritized individuals due to systems of oppression based on ethnic(‐racialized) categorizations, many studies center ethnic(‐racial) identities of minoritized adolescents, whereas majoritized adolescents are seldom included, especially in Europe (Erentaitė et al. 2018). Yet for majoritized students, ethnic identities can also be potential resources, such as for the self‐esteem of white adolescents in the US (Phinney et al. 1997) and when ethnic identification is combined with multiculturalist beliefs for adolescents without migration background in the Netherlands (Verkuyten 2009). For majoritized adolescents, studies suggest that exploring and gaining clarity about their ethnic(‐racial) identities can be beneficial for their self‐esteem and well‐being in the United States (Umaña‐Taylor et al. 2018) and that ethnic identity exploration can be meaningful for majoritized adolescents in Germany through promoting their intercultural competence (Schwarzenthal et al. 2017).

### 2.2 National Identity

In the field of acculturation research, many studies investigated the identification of minoritized youth regarding the national country of residence, oftentimes alongside their ethnic identification with their family heritage culture (e.g., Makarova and Birman 2015; Erentaitė et al. 2018). Consequently, minoritized adolescents are overrepresented while majoritized adolescents are usually not included in these studies.

Studies in Germany indicate that for minoritized students, national identification in terms of identifying exclusively with being German or with the family heritage culture and being German is linked to better academic achievement, and not to psychological well‐being (Baumert et al. 2024; Hannover et al. 2013; Schotte et al. 2018), perhaps due to a strong assimilative climate in school. In contrast, in the United States, national identity development and belonging was linked to favorable outcomes such as lower depressive symptoms, more well‐being and self‐esteem for minoritized adolescents (Meca et al. 2020; Safa et al. 2024).

National identifications can be challenging for minoritized adolescents when they are not accepted as a member of the country of residence, especially in European countries (Gharaei et al. 2024). A persistent challenge for minoritized adolescents' national identity is the exclusionary understanding of who can be German. Such “othering” of minoritized adolescents with a migration history perpetuates exclusion as “eternal migrant” (El‐Tayeb 2014). Denying national identities is a form of subtle discrimination and can pose a developmental risk, for example, through poorer life satisfaction, more school disengagement (Juang, Schwarzenthal et al. 2021) and poorer well‐being (Kiang et al. 2019).

For majoritized students, German identity often remains unaddressed as it is perceived as the norm that does not need explanations or is compared to “other” cultures such as family heritage cultures present in the classroom. Strong national identifications were linked to greater prejudice towards immigrants when majoritized group members had an exclusive understanding of national belonging based on ancestry‐, language‐ and cultural homogeneity (Pehrson et al. 2009). The link between stronger national identification and more prejudice towards immigrants can be weakened, however, when individuals engage in national identity exploration (Spiegler et al. 2022). Students can benefit from exploring and gaining clarity regarding their own understanding of national identity in a more inclusive way, for example, consisting of people who are living in Germany or speak German (Ditlmann and Kopf‐Beck 2019). Conveying an inclusive, pluralistic, and accessible German national identity can be an important opportunity for creating an overarching, unifying identity for majoritized and minoritied adolescents in ethnic‐culturally diverse classrooms (Thijs et al. 2025). To promote inclusive societies, students should reflect on what German identity means for them and for others, how it can include a diversity of ethnic identities, and become aware of being versus not being perceived as the norms. Raising awareness of inclusion and exclusion as well as privileges and disadvantages of minoritized and majoritized youth are opportunities to promote equity in school by encouraging greater acceptance of a diverse society, identifying barriers for belonging of marginalized communities, and recognizing the value of all members of a society.In sum, the findings highlight the importance of understanding ethnic and national identities for both minoritized and majoritzed adolescents and emphasize that social identities like ethnic and national identities of adolescents should be considered as resources for their development, for example, through interventions in school (Hoffman and Umaña‐Taylor 2023).

## 3 Providing Opportunities for Ethnic and National Identity Development in School Through the Identity Project

Interventions can be important ways to promote ethnic identity (Crocetti et al. 2024). Drawing from the benefits of ethnic(‐racial) identities for adolescents' academic achievement and psychological well‐being, a school‐based identity intervention was developed in the United States. The Identity Project (Umaña‐Taylor and Douglass 2017) aims at promoting ethnic(‐racial) identity exploration and resolution, to boost favorable outcomes like global identity coherence, grades, self‐esteem, life satisfaction and outgroup‐orientations and was conceptualized as a universal^4^ intervention for both minoritized and majoritized adolescents. Since 2018, the Identity Project was adapted for the German and other European contexts (Juang et al. 2022).

The goals of the intervention and its' primary focus on engaging with and coming to more clarity about one's ethnic identity remained the same in the German Identity Project. However, to contextualize the understanding of ethnic identity to the German context, one important adaptation concerned explicitly addressing national German identity in an inclusive, pluralistic way, alongside students' ethnic identities. Belonging to and identifying with Germany can be challenging for minoritized adolescents due to a mainstream exclusive interpretation of who is considered “German” (Moffitt et al. 2018). By including a more inclusive understanding of who can belong to and identify with Germany, we tried to prevent “othering” and critiqued the idea of minoritized students as “eternal migrants” (El‐Tayeb 2014). To encourage more open discussion for all students regarding their ethnic identities, it was highlighted that students' engagement with their ethnic identity does not dimish their belongingness to Germany. This inclusive understanding of German national identity was conveyed in discussions of German‐specific examples and experiences that students shared in the classroom, and did not replace the main focus on ethnic(‐racial) identity of the original US intervention.

Based on the theory of change model underlying the intervention, cascading effects are theoretically assumed and empirically supported in the US, whereby the intervention promotes ethnic identity exploration, which then promotes ethnic identity resolution, which in turn boosts favorable outcomes such as greater identity coherence (Umaña‐Taylor and Douglass 2017; Umaña‐Taylor et al. 2018). In Europe, some Identity Project studies suggest the cascades were not distinct. Instead, ethnic identity exploration and resolution changed in parallel (Abdullahi et al. 2024; Hölscher et al. 2024). In other studies, some of the assumed paths were not confirmed, for example, ethnic identity exploration was not boosting resolution (Ceccon et al. 2023) or exploration and resolution were not boosting favorable outcomes (e.g., regarding outgroup and diversity attitudes, Sandberg et al. 2024). However, the intervention effects on students' ethnic identity exploration and resolution were partly replicated in different countries. In Germany, ethnic identity exploration was promoted (Schachner et al. 2024) and was related to more global identity coherence in the intervention group in the pilot study (Juang et al. 2020). In Sweden, ethnic identity exploration and resolution were promoted simultaneously through the intervention (Abdullahi et al. 2024). In Italy, ethnic identity exploration (Ceccon et al. 2023) and ethnic identity resolution (Ceccon, Schachner et al. 2024) were promoted and ethnic identity resolution promoted global identity cohesion (Ceccon, Moscardino et al. 2024). Thus, the intervention is effective in promoting ethnic identity exploration and resolution in Europe, but not necessarily in a step‐wise way.

## 4 Minoritized and Majoritized Adolescent Variations

The process of navigating several social identities like ethnic and national identity into an overall identity may differ for minoritized versus majoritized adolescents. For minoritized adolescents, this navigation may be more stressful as ethnic and national identities can be conflicting in assimilative societies (Branje et al. 2021; Martiny et al. 2017). Based on social identity theory, minoritized adolescents may be confronted with negative group evaluations of their ethnic identities and with restricted access to national identity, which requires greater efforts to integrate both identities into their overall identity and keep a positive sense of self. Furthermore, messages about an exclusive versus inclusive national identity could impact minoritized adolescents' self‐categorization by conveying criteria for group membership (Turner et al. 1987). For majoritized adolescents, ethnic and national identities may be overlapping, referring to the same group, and thus be less likely to be conflictual (Martiny et al. 2017; Thijs et al. 2025). Those with dominant identities also have the luxury of not needing to think much about their identities if they are considered the “norm” (Galliher et al. 2017).

Although the original US studies highlighted the universality of the intervention (Umaña‐Taylor and Douglass 2017), group‐specific differences for minoritized and majoritized students were found with ethnic‐cultural group membership as a moderator. For instance, compared to majoritized students, minoritized students in Italy started higher on ethnic identity resolution before the intervention and after the intervention showed an increase, with this resolution profile being most beneficial for global identity coherence (Ceccon, Moscardino et al. 2024). In the United States, after the intervention, white students reported lower ethnic identity exploration compared to BIPOC students (Wantchekon et al. 2021). Minoritized students in Germany showed different ethnic identity exploration and resolution trajectories compared to majoritized students (Hölscher et al. 2024). While minoritized US students showed more ethnic identity resolution directly after the intervention, majoritized students' ethnic identity resolution did not yet increase. It was only in the long term, after 1 year, that majoritized students gained more clarity about their ethnic identity, and thus attained a similar level as the minoritized students after the intervention (Sladek et al. 2021).

Differences in how interventions like the Identity Project work for minoritized and majoritized adolescents, and differences in how both groups may benefit, have not been the main interest of the previous studies and are not yet extensively examined (Sladek et al. 2021). Of the previous Identity Project studies, none has yet focused on intervention effects on national identity. Also, it remains unclear how both ethnic and national identities contribute to global identity coherence for minoritized and majoritized adolescents. Because there is scattered evidence for how the Identity Project may affect minoritized and majoritized adolescents differently, our tests for variations by these two groups in Germany are exploratory.

## 5 German Context

Since 1957, Germany is a country of immigration (Sachverständigenrat für Integration und Migration 2024a). In 2023, 29.7% of the overall population (and 41% of school‐age children and youth, Sachverständigenrat für Integration und Migration 2024b) either themselves or at least one of their parents has a direct history of migration (German Federal Statistical Office 2024) and half of them are German citizens (Sachverständigenrat für Integration und Migration 2024a). The proportions of people with migration history differs strongly between the federal states: whereas they are 39.4% of the population in Berlin, they make up only 11.4% of the population in Saxony‐Anhalt. Overall, the three most represented heritage countries of people with immigrant decent are Turkey, Poland, and the Russian Federation. Of those immigrants that came to Germany in 2023, the most frequent heritage countries were Ukraine, Syria and Turkey (Sachverständigenrat für Integration und Migration 2024a). Students with migration history compared to those without still score lower in academic performance studies like PISA and Germany has one of the biggest disparity gaps in all OECD countries (Sachverständigenrat für Integration und Migration 2024b). Therefore, it is crucial to investigate school interventions that emphasize supporting adolescents in their identity development, which may serve as resources for other developmental aspects (Hoffman and Umaña‐Taylor 2023).

## 6 The Present Study

In this longitudinal study across three waves of data (T1, T2, and T3), we examined whether the Identity Project implemented after T1 (pretest) data collection promotes adolescents' global identity coherence (less confusion, more synthesis) at T3 through their ethnic and national identity development (exploration and resolution) at T2 differently for ethnic minoritized versus majoritized adolescents. Most studies in Europe tend to either only focus on one aspect (ethnic or national identity) or focus on one group (minoritized, but not majoritized) and have not linked these to global identity coherence. Our study builds on previous studies by including both ethnic and national identities, and testing the model across minoritized and majoritized youth.

We first examine whether the Identity Project implemented after T1 pretest (vs. a control group) promotes students' ethnic and national identity development (exploration and resolution) at T2, and test whether differences for minoritized and majoritized adolescents occur (RQ1). We expect positive effects of the Identity Project on ethnic identity exploration, ethnic identity resolution, national identity exploration and national identity resolution (H1).

We test exploration and resolution at T2 simultaneously, because although the intervention is based on the theory of change, some European replication studies did not support the strict cascades of the intervention predicting exploration and, subsequently, exploration predicting resolution. Based on potential differences concerning questions of belonging to and acceptance of the national German identity and salience of ethnic identity for minoritized and majoritized adolescents, we explore whether the associations of the Identity Project with ethnic and national identity development differ between minoritized and majoritized adolescents.

We further investigate whether students' ethnic and national identity development (exploration and resolution) at T2 mediates the effects of the Identity Project implemented after T1 pretest (vs. a control group) on their global identity confusion and synthesis at T3, and explore whether differences for minoritized and majoritized adolescents occur (RQ2). We expect negative effects of ethnic and national identity development on global identity confusion (decreasing confusion) (H2) and positive effects of ethnic and national identity development on global identity synthesis (increasing synthesis) (H3). Finally, we expect that through the intervention and the subsequent promotion of ethnic and national identity at T2, adolescents develop more global identity coherence at T3 (less confusion, more synthesis) (H4). We will report the direct effects of the intervention on global identity coherence, but expect that this association is explained through the mediators.

Considering the mixed findings from European Identity Project studies regarding the theory of change, we focus on intervention effects on exploration and resolution simultaneously at T2 and explore whether minoritized and majoritized adolescents ethnic and national identity development at T2 will impact their global identity coherence at T3 differently. Although particularly resolution is emphasized to promote global identity coherence in the field, some studies also consider and support the promotive role of exploration (Juang et al. 2020; Safa et al. 2024; Syed et al. 2013) or argue for a bidirectional relation, where global identity coherence can promote exploration and committing to identities but also exploration and commitment may promote global identity coherence (Bogaerts et al. 2019; Crocetti 2017; Schwartz et al. 2009). Thus, we capture how both ethnic and national identity and the developmental processes of exploration and resolution contribute to global identity coherence for minoritized versus majoritized adolescents.

## 7 Methods

### 7.1 Study Design

The Identity Project intervention study was conceptualized with a waitlist‐control‐group‐intervention design. Ethics approval were received from the Berlin Education Senate and the State Board of Education of Saxony‐Anhalt. Classrooms were randomly assigned into either treatment (intervention) or waitlist‐control group. Data were collected at three timepoints: pretest (T1) before any classes participated in the intervention, posttest (T2) 1 week after the treatment classrooms received the eight Identity Project sessions and the control groups received regular teaching content, and follow‐up (T3) 8 weeks after the intervention in the treatment classrooms. After T3, students in the control classrooms received the intervention. Students were given 90 min to fill out the pen‐ and paper‐surveys and received small gifts (e.g., emoji erasers or sweets) as compensation. The data collections were organized and carried out by the research team that were present during the whole time, introduced the surveys, motivated students and answered questions. In most classrooms, teachers were present during the data collection, sitting in the back or observing students behavior, motivating students to fill out the survey or addressing disruptive behavior.

Researchers in Berlin and Saxony‐Anhalt co‐moderated the Identity Project sessions. In every intervention class, one person identifying and perceived as ethnic‐culturally majoritized/white was paired with a ethnic‐culturally minoritized/BIPOC person. Further, one person identifying and perceived as male and the other as female were paired wherever possible. Additionally, one researcher was present during the Identity Project sessions to observe and fill out an implementation fidelity checklist. In the eight Identity Project sessions, strategies (e.g., exchanging with classmates about traditions of their heritage culture groups, interviews with family members, creating a collage with their most relevant ethnic identity elements), contents (e.g., knowledge about Germany's rich migration history, definitions of family heritage culture, stereotypes, discrimination), and discussions (e.g., about students own experiences growing up in Germany or meeting people with different and similar ethnic identities) were provided. Students exchanged their experiences about their ethnic identities with classmates. They were also encouraged to engage with their ethnic identity outside of the classroom, for example through homework involving exploring their neighborhood regarding symbols related to their ethnic identity, or their home and interactions with family members about their ethnic identity.

### 7.2 Positionality Statement

The first author identifies as a BIPOC and light‐skinned woman and as German, is a first‐generation high‐school and college student and comes from a low‐income household. Engaging with diverse ethnic‐cultural and racialized identities and a national (German) identity and belonging are personally meaningful to her. She emphasizes empowering historically marginalized communities, a critical reflection of privileges and raising awareness for inequities in Germany through school, education and research. The second author identifies as Turkish and German. Her research centers understanding how individuals from diverse social groups, national contexts and developmental stages learn about and make sense of diversity and inequity. The third author identifies as a Taiwanese American person with an academic background in developmental psychology and education research from the United States and Germany. She adopts a critical, strength‐based approach in understanding minoritized and majoritized youth identities. The fourth author is a professor with expertise in educational, developmental, and cross‐cultural psychology, comes from a low SES background and identifies as a white German heterosexual woman. In conducting this work, the authors critically reflect on their own backgrounds and assumptions to promote transparency and reflexivity throughout the research process.

### 7.3 Participants

The sample consisted of 581 students (*M*_age_ = 12.83, SD = 0.90, 44% female, 57% with migration history). Data were collected from 26 classrooms, after recruiting six schools in the school years 2018–2019 (100 students), 2019–2020 (97 students; only two waves of data due to COVID‐19‐related school closures) and 2021–2022 (384 students). At the first data collection, participants were 11 (2.8%), 12 (33.9%), 13 (38.7%), 14 (14.1%), 15 (3.3%), and 16 (0.5%) years old, with 6.5% missing information. Data were collected from schools in Berlin (*n* = 223) in all three cohorts, whereas data from schools in Saxony‐Anhalt (*n* = 358) were collected in the final cohort. Data were collected in 7th and 8th grade classrooms, with an expansion to 8th grade classrooms in the final cohort due to COVID‐19‐related unavailability of participating classes. In Germany, the different federal states have different educational systems. While students in Berlin leave primary school after 6th grade, in Saxony‐Anhalt they leave after 4th grade. To highlight the heterogeneity within groups and critically reflect on the categorization of grouping students into ethnic‐culturally minoritized and majoritized adolescents (Vietze et al. 2023), we provide information about the different ethnic‐cultural heritage groups present in the sample as well as information regarding the proportions of mono‐ and bi‐/multicultural self‐identifications in the Table S1.

### 7.4 Measures

Students' reported their age and their migration history as either ethnic‐culturally minoritized (coded as 1 = citizenship other than German and/or at least one of their parents or themselves born outside of Germany) or majoritized (coded as 0 = themselves and parents born in Germany and citizenship German). For the main variables, higher scores on the scale indicate higher agreement. For reliabilities, Cronbach's *α* is reported.

#### 7.4.1 Ethnic Identity

Ethnic identity was measured using the brief version of the Ethnic Identity Scale (EIS‐B, Douglass and Umaña‐Taylor 2015) measuring exploration and resolution. The response scale ranged from 1 = *no, that's not true* to 4 = *yes, that's true*. Three items capture exploration (e.g., “*I have participated in activities through which I was able to learn something about my heritage cultural group*,” *α* for T1/T2/T3 = 0.83/.90/.88) and three items capture resolution (e.g., “*I know what it means to me to belong to my heritage cultural group*,” *α* for T1/T2/T3 = 0.81/.88/.91).

#### 7.4.2 National Identity

National identity was measured using the brief version of the Ethnic Identity Scale (Douglass and Umaña‐Taylor 2015), adapted by Leszczensky and Gräbs Santiago (2014) for use with German youth measuring exploration and resolution. The response scale ranged from 1 = *no, that's not true* to 4 = *yes, that's true*. Three items capture exploration (e.g., “*I have participated in activities through which I was able to learn something about Germany*,” *α* for T1/T2/T3 = 0.72/.78/.86) and three items capture resolution (e.g., “*I know what it means to me to belong to Germany*,” *α* for T1/T2/T3 = 0.84/.87/.91).

#### 7.4.3 Global Identity Coherence

Global identity coherence was measured using the Erikson Psychosocial Stage Inventory (EPSI) Identity subscale (Rosenthal et al. 1981). The response scale ranged from 1 = *no, that's not true* to 5 = *yes, that's true*. Three items captures the subscale of identity confusion, e.g., “*I change my opinion of myself a lot*,” *α* for T1/T2/T3 = 0.59/.66/.66). Five items capture the subscale of identity synthesis (e.g., “*I know what kind of person I am*,” *α* for T1/T2/T3 = 0.67/.77/.80).

### 7.5 Analytical Plan

For missing data, we used Full Information Maximum Likelihood (FIML) as a preferred, highly functional imputation method (Liu and Sriutaisuk 2021). We used SPSS 29 to report the sample demographics, the bivariate correlations, and M*plus* for confirmatory factor analyses (CFAs), measurement invariance and multigroup mediation analyses. For preliminary analysis, we conducted multigroup CFAs of ethnic and national identity scales with exploration and resolution to examine the factor structure for minoritized and majoritized adolescents and ensure the comparability of the measures across groups. Then, Pearson's correlation coefficients were reviewed for the two groups (minoritized vs. majoritized) separately.

**Figure 1:**
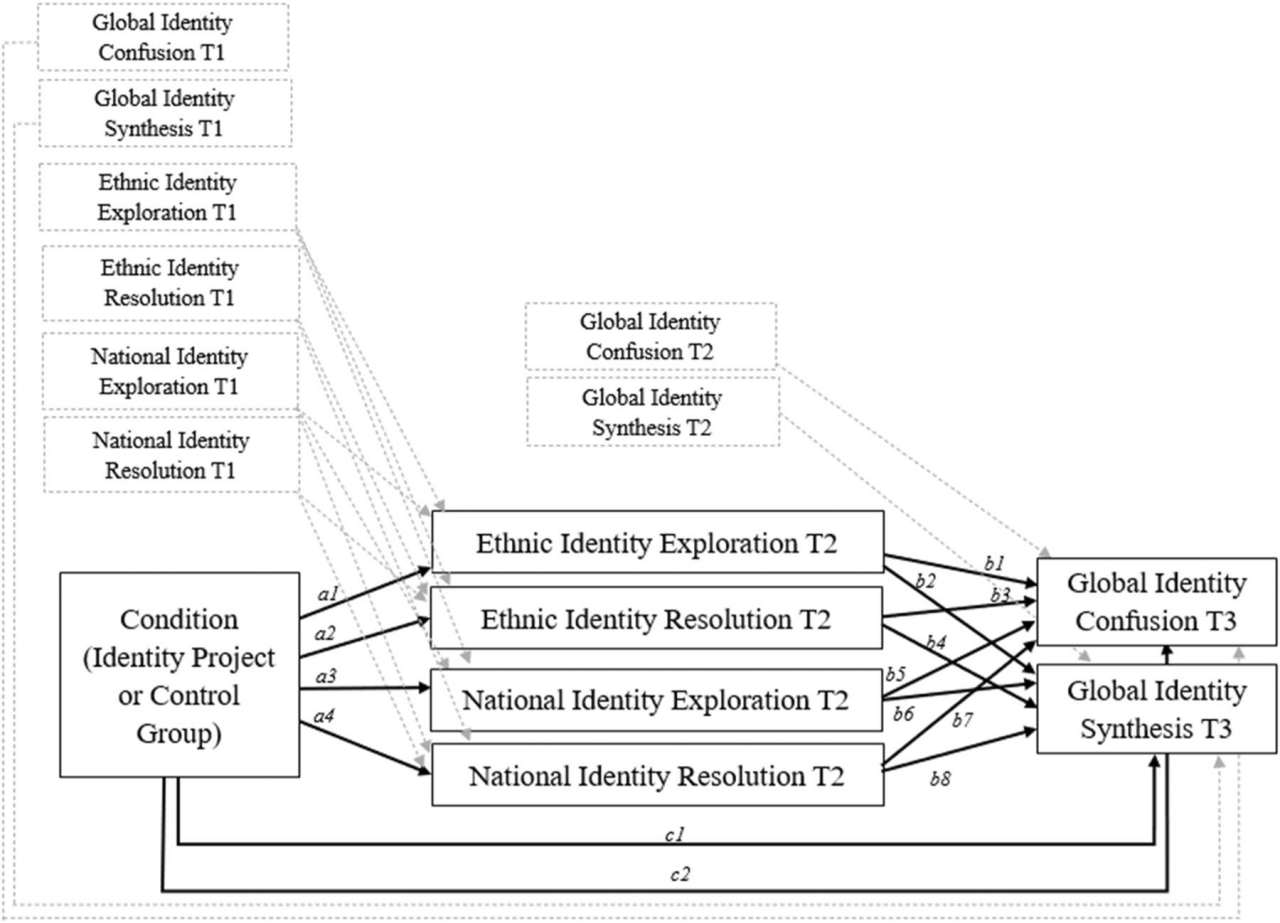
Multigroup parallel mediation model. Conceptual Diagram with covariates, representing the direct and indirect effects of the condition (treatment vs. control group) on T3 global identity coherence (confusion and synthesis) through T2 ethnic and national identity development (exploration and resolution). Multigroup analyses are based on ethnic‐culturally minoritized versus majoritized students.

The full conceptual model is depicted in Figure 1. To test RQ1 and RQ2, a multigroup (comparing minoritized vs. majoritized groups) mediation model was used examining whether ethnic identity (T2 ethnic identity exploration and T2 ethnic identity resolution) and national identity (T2 national identity exploration and T2 national identity resolution) mediated the relationships between participation in the intervention (vs. a control group) and global identity coherence (T3 global identity confusion and T3 global identity synthesis as outcome variables), using the Model Indirect command in M*plus*.

For the accuracy of regression estimates, we included the previous timepoints of the study variables to the model as covariates (MacKinnon et al. 2007). We specified the correlations of the mediators, assuming all T2 ethnic and national identity variables correlate with each other. The correlations of the exogenous predictors (T1 ethnic and national identity development, T1 and T2 global identity coherence) are considered in M*plus* by default.

We first estimated an unconstrained multigroup model, where all path coefficients are allowed to vary freely across minoritized and majoritized youth. We then estimated a fully constrained model, where all path coefficients are estimated to be equal. To test for group differences (minoritized vs. majoritized youth), we used the Model Test command in M*plus*. Invariance of nested models (measurement invariance and invariance of the unconstrained and constrained models) are evaluated based on *χ* ^2^ difference tests and recommended thresholds of ΔCFI ≤ 0.005 paired with ΔRMSEA ≤ 0.010 or ΔSRMR ≤ 0.025 for metric and ΔSRMR ≤ 0.005 for scalar invariance for a sample size of < 300 and unequal group sizes (Chen 2007).

We further explored differences of individual paths for minoritized versus majoritized adolescents through additional sequential testing using Wald tests to accurately specify our main model. The Wald test works well with sample sizes similar to the sample size of the present study and with unequal sizes of compared groups (Woods et al. 2013). This is not the standard procedure for multigroup mediation analyses in case invariance is supported based on the mentioned thresholds of the global test. However, it can be reasonable if there is a theoretical and empirical basis to assume differences of individual paths for two groups (Byrne 2010; Kline 2016). For minoritized versus majoritized adolescents, there is evidence to suspect differences regarding their ethnic and national identity development (e.g., Ceccon, Moscardino et al. 2024; Froehlich et al. 2020; Hölscher et al. 2024). Subtle but important differences of single paths can remain undetected and masked with the global omnibus test (Oberski 2014), especially in complex models and with relatively small subgroups (MacCallum et al. 2006; Shi et al. 2019). Forcing invariance where it does not exist can lead to biased or misleading parameter estimates (Edwards et al. 2018; Oberski 2014; Shi et al. 2019). Therefore, for group comparisons, allowing noninvariant parameters to vary can be reasonable if parsimony and accuracy of the multigroup model are balanced (Byrne 2010; Hsiao and Lai 2018; Oberski 2014).

We tested differences of the a1–a4 paths by testing ethnic identity exploration first (a1), then ethnic identity resolution (a2), followed by national identity exploration (a3) and national identity resolution (a4), by freeing each individual path while keeping all other paths constrained as equal across the two groups. We tested ethnic identity paths first as there are strong theoretical arguments for why this aspect may differ between minoritzed and majoritized youth. Ethnic identity may be more central than national identity for minoritized youth (Froehlich et al. 2020), potentially due to societal phenomenon such as “othering,” not being perceived as the ethnic‐cultural norm, and heightened salience of minority group membership (Umaña‐Taylor et al. 2014). Empirically, studies report greater ethnic identity exploration (Wantchekon et al. 2021), and stronger resolution for minoritized youth (Sladek et al. 2021) compared to majority youth or differences in the ethnic identity trajectories for minoritized versus majoritized youth (Hölscher et al. 2024). We tested ethnic exploration before resolution as the intervention's main aim was to prompt exploration. We then tested national identity exploration and resolution. Again, exploration was tested before resolution as the intervention was meant to prompt exploration. National identity was not the main target of the intervention, so we assumed that compared to ethnic identity it may be less likely to change.

To determine the model fit of our multigroup mediation models, we followed Hu and Bentler (1999) with CFI values ranging from 0.90 to 0.95, RMSEA values < 0.08; and the SRMR value below 0.06. We pursue a more flexible interpretation of the TLI, ideally > 0.90 but acceptable if > 0.80, if other fit parameters indicate a good fit, because the TLI is considered as underestimating fit for samples of < 200 (e.g., Tabachnick and Fidell 2007). To assess the significance of the indirect effects and provide more robust estimates, we report confidence intervals based on bias‐corrected bootstrapping with 5000 sample draws (MacKinnon et al. 2004). The mediating effect is considered significant, if the 95% CI of the indirect effect does not include zero (Hayes 2022; MacKinnon et al. 2004).

## 8 Results

### 8.1 Descriptives and Preliminary Analyses

#### 8.1.1 Attrition and Missing Data

There was attrition in the longitudinal data, with 15.0%–16.7% missing for T1, 19.9%–20.8% for T2 and 31.7%–32.4% missing for T3. Little (1988) missing completely at random test indicated that for all three timepoints, missing values were missing completely at random for both minoritized and majoritized students (T1_minoritized_ *χ* ^2^ = 19.817, *df* = 25, *p* = 0.756; T1_majoritized_ *χ* ^2^ = 17.924, *df* = 13, *p* = 0.160; T2_minoritized_ *χ* ^2^ = 22.949, *df* = 18, *p* = 0.193; T2_majoritized_ *χ* ^2^ = 32.393, *df* = 23, *p* = 0.092; T3_minoritized_ *χ* ^2^ = 16.911, *df* = 16, *p* = 0.391; T3_majoritized_ *χ* ^2^ = 8.318, *df* = 12, *p* = 0.760).

#### 8.1.2 Confirmatory Factor Analyses

**Figure 2:**
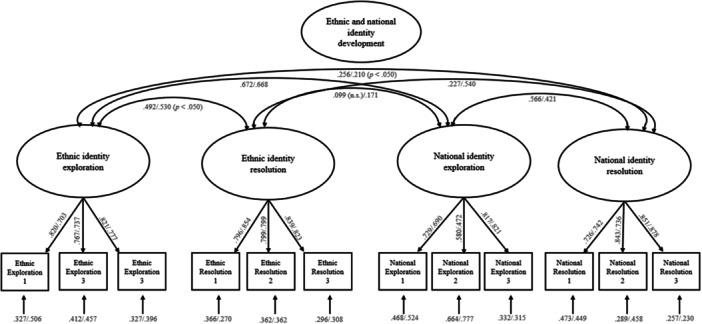
Confirmatory factor analyses for ethnic and national identity development at timepoint 1. *N* = 581 (minoritized adolescents *n* = 331, majoritized adolescents *n* = 250). Multigroup analyses for minoritized/majoritized students, with coefficients for minoritized students before and for majoritized students after the slash. Coefficients are significant *p* < 0.001 if not highlighted otherwise.

We conducted CFAs to test the factor structure of ethnic and national identity. The four‐factor‐model showed a good model fit for minoritized (*χ* ^2^/*df* (48) = 93.507, *p* < 0.0001, CFI = 0.970, TLI = 0.959, RMSEA = 0.058, [90% CI = 0.040, 0.075], SRMR = 0.029) and majoritized (*χ* ^*2*^/*df* (48) = 95.892, *p* = 0.0000, CFI = 0.955, TLI = 0.938, RMSEA = 0.068, 90% CI = [0.048, 0.088], SRMR = 0.045) adolescents. The CFAs showed the national identity exploration item 2 (“I have read books/magazines/newspapers or something else to learn something about Germany”) had low factor loadings (minoritized = 0.580; majoritized = 0.472) and strong residual variances (*σ* ^2^_minoritized_ = 0.664; *σ* ^2^_majoritized_ = 0.777) for both groups (see Figure 2 for standardized factor loadings). As the national identity exploration scale consists of three items in total, we decided to keep item 2 for the scale measurement. There were differences regarding a stronger latent correlation of the factors of ethnic identity resolution and national identity resolution for majoritized students (*r* = 0.540) compared to minoritized students (*r* = 0.227) (*F*(3, 478) = 17.896, *p* < 0.001, *z*‐standardized regression coefficient for majoritized students*T1 national identity resolution on T1 ethnic identity resolution = 0.169, 95% CI [0.063; 0.276]). Multigroup and longitudinal measurement invariance test results are shown in Tables S6–S14 in the Supporting Information. Metric invariance was supported for all measures across groups and across time. Scalar invariance was supported for ethnic identity across groups as well as across time for national identity exploration, national identity resolution, ethnic identity exploration, ethnic identity resolution, and global identity confusion. Scalar invariance was not supported for national identity and global identity coherence across groups and for global identity synthesis across time.

#### 8.1.3 Descriptives and Correlation Analyses

In Table 1, descriptives (means, SD) and bivariate correlations are reported separately for minoritized and majoritized students. On the mean level, majoritized students started lower on ethnic identity exploration at T1 (*M* = 2.04, *t*(484) = −6.291, *p* < 0.001, mean difference = −0.550, 95% CI [−0.772; −0.378]) and ethnic identity resolution at T1 (*M* = 2.80, *t*(485) = −8.545, *p* < 0.001, mean difference = −0.628, 95% CI [−0.772; −0.485]) than minoritized students (*M* = 2.50/3.43). For T1 global identity confusion, majoritized students scored higher (*M* = 2.99, *t*(494) = 2.224, *p* = 0.027; mean difference = 0.226, 95% CI [0.026; 0.425]) than minoritized students (*M* = 2.76). Global identity confusion decreased from T1–T3 for minoritized (*M*_*T3*_ = 2.60, *t*(174) = 2.419, *p* = 0.017) and majoritized (*M*_*T3*_ = 2.72, *t*(162) = 4.420, *p* < 0.001) students. For global identity synthesis, both groups started similarly high at T1 (*t*(494) = −0.361, *p* = 0.718; mean difference = −0.026, 95% CI [−0.170; 0.117]) and scores decreased from T1–T3 for minoritized adolescents (*M* = 4.15/3.98, *t*(174) = 3.186, *p* = 0.002) and majoritized adolescents (*M* = 4.12/3.67, *t*(162) = 5.831, *p* < 0.001).

**Table 1:** Bivariate correlations and descriptives.

Variable	T1 EI exploration	T1 EI resolution	T1 NI exploration	T1 NI resolution	T1 GI confusion	T1 GI synthesis	T2 EI exploration	T2 EI resolution	T2 NI exploration	T2 NI resolution	T2 GI confusion	T2 GI synthesis	T3 EI exploration	T3 EI resolution	T3 NI exploration	T3 NI resolution	T3 GI confusion	T3 GI synthesis
1. T1 EI exploration	—	0.438***	0.514***	0.165*	−0.034	0.118	0.411***	0.168*	0.369***	0.077	0.028	0.046	0.419***	0.142	0.398***	0.154*	−0.016	0.126
2. T1 EI resolution	0.424***	—	0.120	0.465***	−0.165*	0.310***	0.335***	0.374***	0.179*	0.301***	−0.066	0.208**	0.226**	0.468***	0.190*	0.401***	−0.094	0.277***
3. T1 NI exploration	0.543***	0.078	—	0.331***	0.091	−0.044	0.317***	0.046	0.442***	0.165*	0.070	−0.051	0.265***	0.022	0.337***	0.108	−0.042	0.041
4. T1 NI resolution	0.197**	0.193**	0.445***	—	0.019	0.224**	0.197*	0.329***	0.230**	0.461***	0.057	0.221**	0.061	0.412***	0.083	0.471***	−0.140	0.192*
5. T1 GI confusion	0.096	−0.025	0.113	0.067	—	−0.280***	0.023	0.030	0.157*	0.089	0.564***	−0.302***	−0.024	−0.029	0.019	−0.018	0.512***	−0.315***
6. T1 GI synthesis	0.216***	0.298***	0.193**	0.242***	−0.016	—	0.038	0.181*	−0.028	0.148	−0.296***	0.560***	0.106	0.293***	0.080	0.302***	−0.318***	0.437***
7. T2 EI exploration	0.571***	0.119	0.389***	0.053	0.013	0.075	—	0.496***	0.636***	0.220*	0.115	0.149*	0.500***	0.190*	0.457***	0.226**	0.021	0.089
8. T2 EI resolution	0.250***	0.368***	0.073	0.118	−0.024	0.159*	0.321***	—	0.229**	0.591***	−0.061	0.355***	0.187*	0.432***	0.211**	0.390***	−0.107	0.231**
9. T2 NI exploration	0.322***	0.015	0.578***	0.281***	0.030	0.048	0.440***	0.135*	—	0.314***	0.163*	0.097	0.451***	0.088	0.540***	0.204**	−0.016	0.033
10. T2 NI resolution	0.106	0.130	0.340***	0.576**	0.027	0.120	0.023	0.153*	.444***	—	0.001	0.343***	0.078	0.414***	0.288***	0.554***	−0.080	0.221**
11. T2 GI confusion	0.046	−0.135*	0.079	0.021	0.471***	−0.137*	0.054	−0.082	0.029	−0.015	—	−0.330***	0.109	−0.034	0.122	−0.018	0.536***	−0.301***
12. T2 GI synthesis	0.084	0.193**	0.050	0.138*	−0.214***	0.306***	0.153*	0.365***	0.134*	0.283***	−0.233***	—	0.079	0.261***	0.100	0.278***	−0.279***	0.413***
13. T3 EI exploration	0.557***	0.201**	0.433***	0.136	0.222**	0.055	0.712***	0.349***	0.406***	0.084	0.188**	0.161*	—	0.377***	0.696***	0.184*	0.011	0.180*
14. T3 EI resolution	0.260***	0.507***	0.057	0.217**	0.044	0.242**	0.193*	0.521***	0.056	0.169*	−0.081	0.313***	0.360***	—	0.277**	0.698***	−0.068	0.494***
15. T3 NI exploration	0.443***	−0.019	0.680***	0.259***	0.118	0.050	0.379***	0.056	0.633***	0.321***	0.104	0.077	0.451***	0.060	—	0.286***	0.032	0.066
16. T3 NI resolution	0.106	0.210**	0.308***	0.552***	0.012	0.146	−0.011	0.167*	0.356***	0.603***	−0.099	0.258***	0.126	0.316***	0.471***	—	−0.083	0.385***
17. T3 GI confusion	0.105	−0.244**	0.185*	0.025	0.479***	−0.205**	0.159*	−0.094	0.099	−0.077	0.652***	−0.153*	0.197**	−0.036	0.186**	−0.039	—	−0.358***
18. T3 GI synthesis	0.124	0.341***	0.118	0.126	−0.110	0.315***	−0.005	0.247**	0.082	0.315***	−0.176*	0.573***	0.153*	0.422***	0.041	0.360***	−0.106	—
Minoritized students																		
*M (SD)*	2.50 (1.05)	3.43 (0.75)	2.21 (0.92)	2.81 (0.84)	2.76 (1.12)	4.15 (0.81)	2.71 (1.03)	3.51 (0.69)	2.19 (0.89)	2.77 (0.86)	2.54 (1.06)	4.01 (0.81)	2.60 (1.04)	3.45 (0.71)	2.13 (0.94)	2.79 (0.90)	2.60 (1.03)	3.98 (0.83)
Majoritized students																		
*M (SD)*	2.04 (0.83)	2.80 (0.87)	2.26 (0.76)	2.92 (0.75)	2.99 (1.12)	4.12 (0.80)	2.08 (0.98)	2.85 (0.89)	2.17 (0.87)	2.84 (0.78)	2.74 (1.00)	3.73 (0.92)	2.00 (0.90)	2.86 (0.91)	2.14 (0.90)	2.89 (0.88)	2.72 (0.97)	3.67 (0.85)

*Note: N*_minoritized_ = 331, *N*_majoritized_ = 250. Correlations for minoritized students below and for majoritized students above the diagonal.

Abbreviations: EI = ethnic identity, GI = global identity coherence, NI = national identity.

* *p* < 0.05

** *p* < 0.01

*** *p* < 0.0.

The correlations between the mediators (ethnic and national identity exploration and resolution) were all positive and ranged from weak to medium. Adolescents' global identity synthesis at T3 was correlated with more ethnic and national identity resolution (but not exploration) at T2 for majoritized (*r* = 0.231, *p* = 0.003*/r* = 0.221, *p* = 0.004) and minoritized students (*r* = 0.247, *p* = 0.001/*r* = 315, *p* < 0.001). Global identity synthesis at T3 was positively correlated with previous same‐scale measurements for majoritized (*r*_T1_ = 0.437, *p* < 0.001/*r*_T2_ = 0.413, *p* < 0.001) and minoritized (*r*_T1_ = 0.315, *p* < 0.001/*r*_T2_ = 0.573, *p* < 0.001) students. Interestingly, global identity synthesis at T3 was significantly correlated with less confusion at T3 for majoritized students (*r* = −0.358, *p* < 0.001) but not for minoritized students (*r* = −0.106, *p* = 0.125) (*F*(3, 397) = 7.527, *p* = 0.006, *z*‐standardized regression coefficient for majoritized students*T3 global identity confusion on T3 global identity synthesis = −0.225, 95% CI [−0.386; −0.064]). Global identity confusion at T3 was positively correlated with previous same‐scale measurements for majoritized (*r*_T1_ = 0.512, *p* < 0.001/*r*_T2_ = 0.536, *p* < 0.001) and minoritized (*r*_T1_ = 0.479, *p* < 0.001/*r*_T2_ = 0.652, *p* < 0.001) students.

### 8.2 Main Analyses

**Figure 3:**
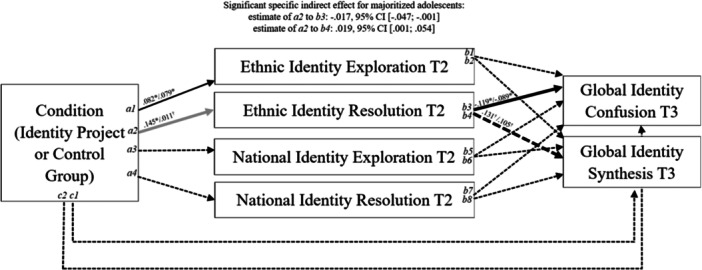
Significant path coefficients and mediation effects for minoritized versus majoritized students. *N* = 581 (minoritized adolescents *n* = 331, majoritized adolescents *n *= 250). Standardized path coefficients are reported for majoritized/minoritized adolescents, with estimates for majoritized adolescents before and for minoritized adolescents after the slash. Dashed line indicates nonsignificant path for both groups. Light gray line indicates significant path for one of the groups. Solid black line indicates significant path for both groups. Bold lines indicate significant indirect effects. Dashed bold lines indicate a significant indirect effect despite insignificant a path and/or b path. The results derive from the conceptual model shown in Figure 1, including covariates and correlations. ^†^ *p* > 0.05, *p < 0.05, **p < 0.01, ***p < 0.001.

The model results for the direct and indirect effects and correlations among the mediator and outcome variables are reported in Table 2. Significant path coefficients and indirect effects are depicted in Figure 3. Standardized estimates and confidence intervals are reported. We first compared the unconstrained model (*χ* ^*2*^ */df* (84) = 171.136, *p* = 0.0000, CFI = 0.925, TLI = 0.877, RMSEA = 0.060, 90% CI = [0.047, 0.073], SRMR = 0.053) to a fully constrained model where all paths are constrained to be equal for minoritized and majoritized adolescents (*χ* ^*2*^ */df* (98) = 184.417, *p* = 0.0000, CFI = 0.926, TLI = 0.896, RMSEA = 0.055, 90% CI = [0.043, 0.067], SRMR = 0.054). The fully constrained model did not significantly differ compared to unconstrained model (*χ* ^2^Δ(14) = 13.281, *p* = 0.505, ΔCFI = 0.001, ΔRMSEA = −0.005, ΔSRMR = 0.001). This indicates invariance across the two groups.

**Table 2:** Direct effects, indirect effects, and correlations of the independent variable, the mediator variables and the outcome variables of the final model.

	Minoritized adolescents		Majoritized adolescents	
Direct effects	*β* (SE)	*p* value	*β* (SE)	*p* value
Path A				
Intervention (vs. control)→EI exploration *(a1)*	**0.079 (0.040)**	**0.046**	**0.082 (0.041)**	**0.046**
Intervention (vs. control)→EI resolution T2 *(a2)*	0.011 (0.057)	0.849	**0.145 (0.057)**	**0.011**
Intervention (vs. control)→NI exploration T2 *(a3)*	0.038 (0.041)	0.348	0.038 (0.041)	0.348
Intervention (vs. control)→NI resolution T2 *(a4)*	0.065 (0.039)	0.099	0.070 (0.042)	0.095
Path B				
EI exploration T2→GI confusion T3 *(b1)*	0.104 (0.059)	0.077	0.108 (0.061)	0.076
EI exploration T2→GI synthesis T3 *(b2)*	–0.111 (0.066)	0.096	–0.107 (0.064)	0.097
EI resolution T2→GI confusion T3 *(b3)*	**–0.089 (0.044)**	**0.044**	**–0.119 (0.058)**	**0.042**
EI resolution T2→GI synthesis T3 *(b4)*	0.105 (0.054)	0.054	0.131 (0.068)	0.052
NI exploration T2→GI confusion T3 *(b5)*	–0.058 (0.055)	0.295	–0.061 (0.059)	0.301
NI exploration T2→GI synthesis T3 *(b6)*	0.024 (0.062)	0.696	0.024 (0.061)	0.695
NI resolution T2→GI confusion T3 *(b7)*	–0.038 (0.054)	0.482	–0.037 (0.053)	0.483
NI resolution T2→GI synthesis T3 *(b8)*	0.056 (0.063)	0.376	–0.051 (0.057)	0.369
Path C				
Intervention (vs. control)→GI synthesis T3 *(c1)*	–0.035 (0.044)	0.422	–0.035 (0.044)	0.420
Intervention (vs. control)→GI confusion T3 *(c1)*	–0.053 (0.039)	0.169	–0.057 (0.041)	0.166

	Minoritized adolescents		Majoritized adolescents	
Indirect effects	*β*	BC CI (LL, UL)	*β*	BC CI (LL, UL)
Condition→mediator→outcome				
Intervention (vs. control)→EI exploration T2→GI confusion T3 *(a1*→*b1)*	0.008	[0.000, 0.028]	0.009	[0.000, 0.030]
Intervention (vs. control)→EI exploration T2→GI synthesis T3 *(a1*→*b2)*	–0.009	[–0.030, 0.009]	–0.009	[–0.030, 0.000]
Intervention (vs. control)→EI resolution T2→GI confusion T3 *(a2*→*b3)*	–0.001	[–0.015, 0.009]	**–0.017**	**[–0.047, –0.001]**
Intervention (vs. control) → EI resolution T2 → GI synthesis T3 *(a2* → *b4)*	0.001	[–0.012, 0.016]	**0.019**	**[0.001, 0.054]**
Intervention (vs. control)→NI exploration T2→GI confusion T3 *(a3*→*b5)*	–0.002	[–0.016, 0.002]	–0.002	[–0.017, 0.002]
Intervention (vs. control)→NI exploration T2→GI synthesis T3 *(a3*→*b6)*	0.001	[–0.003, 0.014]	0.001	[–0.003, 0.014]
Intervention (vs. control)→NI resolution T2→GI confusion T3 *(a4*→*b7)*	–0.002	[–0.016, 0.003]	–0.003	[–0.017, 0.003]
Intervention (vs. control)→NI resolution T2→GI synthesis T3 *(a4*→*b8)*	0.004	[–0.003, 0.021]	0.004	[–0.003, 0.020]

	Minoritized adolescents		Majoritized adolescents	
Correlations	*r* (SE)	*p* value	*r* (SE)	*p* value
Correlations among the mediators				
EI exploration T2↔EI resolution T2 (*mediator↔mediato*r *2)*	**0.268 (0.063)**	**0.000**	**0.491 (0.063)**	**0.000**
EI resolution T2↔NI exploration T2 *(mediator 2↔mediator 3)*	0.111 (0.060)	0.064	**0.215 (0.087)**	**0.013**
NI exploration T2↔NI resolution T2 *(mediator 3↔mediato*r *4)*	**0.350 (0.065)**	**0.000**	**0.290 (0.076)**	**0.000**
NI resolution T2↔EI exploration T2 *(mediator 4↔mediator 1)*	–0.059 (0.073)	0.416	0.166 (0.086)	0.054
NI exploration T2↔EI exploration T2 (*mediator 3↔\mediator 1*)	**0.319 (0.066)**	**0.000**	**0.550 (0.073)**	**0.000**
EI resolution T2↔NI resolution T2 (m*ediator 2↔mediator* 4)	0.118 (0.081)	0.146	**0.525 (0.086)**	**0.000**
Correlations among the outcomes				
GI synthesis T3↔GI confusion T3 (o*utcome 2↔outcome 1)*	0.072 (0.107)	0.498	–0.174 (0.098)	0.075

*Note: N* = 581 (minoritized adolescents *n* = 331, majoritized adolescents *n* = 250). Standardized coefficients are reported. Significant direct and indirect effects and correlation coefficients are highlighted in bold.

Abbreviations: BC CI, 95% bias‐corrected confidence intervals; EI, ethnic identity; GI, global identity; LL, lower limit; NI, national identity; SE, standard error; T2, timepoint 2 (1 week posttreatment in the intervention classrooms); T3, timepoint 3 (8 weeks posttreatment in the intervention classrooms); UL, upper limit.

The explorative analyses of differences of individual paths, however, showed that the intervention versus control group was differentially related to ethnic identity resolution at T2 for minoritized and majoritized adolescents (Wald *χ* ^2^ (1, 581) = 4.227, *p* = 0.042), but no other individual paths of our multigroup model. As a consequence, the path a2 (See Figure 1) was released to vary across minoritized/majoritized adolescents^5^. The partly constrained model did not significantly differ compared to the unconstrained model (*χ* ^2^Δ(13) = 9.182, *p* = 0.759, ΔCFI = 0.004, ΔRMSEA = −0.006, ΔSRMR = −0.001) and also showed a better model fit. The model fit of the partly constrained model (*χ* ^2^ */df* (97) = 180.318, *p* = 0.0000, CFI = 0.929, TLI = 0.898, RMSEA = 0.054, 90% CI = [0.042, 0.067], SRMR = 0.054) of our multigroup mediation model was acceptable concerning the complexity of the model and relatively small subsample sizes (majoritized_Intervention_ *n* = 134, minoritized_Intervention_ *n* = 163, majoritized_Control_ *n* = 116, minoritized_Control_ *n* = 168). Hence, the partly constrained model was retained and treated as the final model.

For RQ1, we examine the effects of the Identity Project (vs. control group) on students' ethnic (path a1 and a2) and national identity (path a3 and a4) exploration and resolution. The intervention promoted ethnic identity exploration of minoritized (*β* = 0.079, *p* = 0.046) and majoritized (*β* = 0.082, *p* = 0.046) students, and ethnic identity resolution at T2 for majoritized students (*β* = 0.145, *p* = 0.011). We found no other intervention effects for either of the groups on national identity exploration and resolution at T2. Thus H1 is partly supported.

To address RQ2, we report whether students ethnic and national identity development (exploration and resolution) at T2 are associated with global identity confusion at T3 (H2) and synthesis at T3 (H3) and whether they mediate the effects of the intervention on adolescents' T3 global identity confusion (paths b1, b3, b5, b7) and synthesis (paths b2, b4, b6, b8) (H4). For both minoritized adolescents (*β* = ‐.089, *p* = 0.044) and majoritized adolescents (*β* = −0.119, *p* = 0.042), ethnic identity resolution at T2 negatively predicted global identity confusion at T3. No other effects of national or ethnic identity development on global identity confusion or synthesis were identified. Hence H2 is partly supported, while H3 is rejected.

There were no direct effects of the Identity Project on neither minoritized nor majoritized adolescents' global identity synthesis (c1 and c2 path). Regarding the mediated effects, the intervention promoted ethnic identity resolution at T2, which in turn related to lower global identity confusion at T3 (*β* = −0.017, 95% (CI: −0.047; −0.001)) and more global identity synthesis at T3 (*β* = 0.019, 95% (CI: 0.001; −0.054)), but only for majoritized adolescents. We found no other significant indirect effects. Consequently, H4 is partly supported. In total, our model explained 49.3% of the variance of global identity confusion and 37.1% of global identity synthesis at T3 for minoritized adolescents. For majoritized adolescents, 37.7% of the variance of global identity confusion and 20.1% of global identity synthesis at T3 were explained.

### 8.3 Additional Analyses

In addition to analyses addressing the research questions in the multigroup mediation model, interesting findings concerning the associations with the exogenous predictors and the mediators are shared in a footnote.^6^ To test for the robustness of our results, we tested four alternative models: (1) analyses without using FIML, (2) including covariates of age, region, and cohort, (3) including the whole sample (not by minoritized vs. majoritized adolescents), and (4) including the whole sample testing a limited theory of change.^7^ In the first and second alternate model, similar to the main results, the intervention promoted greater ethnic identity exploration for both groups and ethnic identity resolution only for majoritized adolescents. One difference is that in the first alternate model, the mediation effects of ethnic identity resolution showed for global identity synthesis but not confusion for majoritized adolescents and of ethnic identity exploration predicting less global identity synthesis for both groups. In the second alternate model, results replicated the main mediation results in terms of the intervention reducing global identity confusion through ethnic identity resolution for majoritized youth. It differed so that ethnic identity resolution did not predict global identity synthesis for majoritized adolescents and in terms of showing increased global identity confusion for both groups through ethnic identity exploration. In the third alternate model, similar to the main results, ethnic identity resolution was related to less global identity confusion but for adolescents in the intervention and control condition. Unlike the reported main results, ethnic identity exploration related to more global identity confusion and there were no intervention effects and no mediation. In the fourth alternate model, there were no intervention effects (again with the whole group, similar to the third alternate model), but the results showed support for the theory of change in that ethnic identity exploration at T2 positively promoted ethnic identity resolution at T2 which positively predicted global identity synthesis at T3. The alternative models generally align with what is reported in the main text. They showed variations in their results but all at least partly supported the findings reported in the main analyses. The findings support the subgroup‐specific analyses (minoritized vs. majoritized adolescents), as otherwise relevant subgroup variations and intervention effects may be obscured. All alternate models showed a poorer fit than the reported model for the main analyses, supporting its selection as the final model given the good fit as well as the theoretical and empirical basis. For more details, please see Figures S2–S5.

## 9 Discussion

The purpose of the study was to examine how ethnic and national identities contribute to minoritized and majoritized adolescents' global identity coherence, through exploring and gaining more clarity about those identities in a school intervention. Complementing previous studies on the Identity Project (e.g., Ceccon, Moscardino et al. 2024; Umaña‐Taylor et al. 2018), ethnic and national identifications in Germany (e.g., Baumert et al. 2024), and ethnic identity development in Germany (e.g., Juang et al. 2020; Schachner et al. 2024), we explored differences for minoritized and majoritized students and included national identity development. Examining questions of ethnic and national identity is relevant in diverse contexts like Germany, where adolescents are confronted with questions of belonging to and identifications with ethnic and national groups. Importantly, the experiences of minoritized and majoritized adolescents in those contexts can differ, and could lead to differences in their ethnic and national identity development when participating in the Identity Project.

The first research question (RQ1) focused on whether the Identity Project can promote students' ethnic and national identities. The intervention indeed supported the exploration of ethnic identity at T2 for both minoritized and majoritized adolescents, which supports one of the main aims of the intervention and the first cascade of the theory of change (Umaña‐Taylor and Douglass 2017). Furthermore, majoritized students gained more clarity about their ethnic identity through the intervention. Minoritized adolescents were already starting very high on ethnic identity resolution at the baseline, suggesting that they may have already explored and gained some clarity regarding their ethnic identities before the intervention through engaging in ethnic(‐racial) socialization with parents, peers, or media (Aral et al. 2022; Sladek et al. 2021; Wang et al. 2023). Thus, potential ceiling effects of ethnic identity resolution could have prohibited strong increases of minoritized youth in the intervention group in their ethnic identity resolution (Sladek et al. 2021). For majoritized adolescents in the German Identity Project, the strategies and discussion of the intervention were helpful to come to more clarity regarding their ethnic identity, and thus matched to the higher levels of their minoritized peers. We find these effects already directly after the intervention, while it took white youth in the US intervention much longer (Sladek et al. 2021). The shorter time‐frame in Germany for majoritized youth to show ethnic identity resolution could be due to the context where they are perceived as the ethnic‐cultural norm. Perhaps the Identity Project intervention was the first time they were actually encouraged to actively engage with and reflect on their ethnic identity, rather than remaining unaddressed and unchallenged in their own families, communities, and in school. Importantly, our findings show that the tempo of intervention effects may differ depending on the particular sociohistorical context.

The intervention did not promote students' national identity development, perhaps because it was not designed to target national identity specifically. Even though we expanded on the discussions of ethnic identity by including dicussions of national identity, this may not have been enough to promote national identity exploration and resolution. Mastrotheodoros et al. (2021) emphasize the importance of the sociocultural climate in immigration societies in ensuring that minoritized adolescents ethnic and national identity are not conflicting—especially in assimilationist and exclusive contexts. Although the intervention facilitators tried to convey an inclusive, pluralistic understanding of who can be German and what this can mean, this may have been ineffective because the exclusive understanding of who a *real* German is (Moffitt et al. 2018), can still be present in contexts outside the Identity Project classrooms. We also interpret that potentially, when students' exclusive understandings of who can be German are challenged, the distinctiveness of this self‐categorization could be questioned (Turner et al. 1987). This could make the national identity less relevant to majoritized students, and harder to explore for both majoritized and minoritized adolescents. For majoritized students, the question may arise: If many people with different ethnic identities have a German national identity, what does this mean for my German national identity? We also wonder whether the negative associations with Germany that are discussed in the Identity Project, like racism of minoritized groups in Germany or the Nazi history, can hinder majoritized students to engage more with their national identity as this information could prompt negative emotions and feelings of guilt (Juang, Moffitt et al. 2021).

The CFA and the correlations of the mediators and associations with the exogenous predictors indicate that for majoritized students, their ethnic and national identities are more overlapping than for minoritized students (Martiny et al. 2017; Thijs et al. 2025), supporting the idea that in Germany, national identities may be more closely restricted to the ethnic German majority (Moffitt et al. 2018). Nonetheless, our findings show that ethnic and national identites are distinct social identities for minoritized (Froehlich et al. 2020; Mastrotheodoros et al. 2021) and also majoritized adolescents. For adolescents with a German family heritage culture and who grew up in Germany, it seems logical that their ethnic and national identity can overlap. However, some students could have considered their family heritage identity as belonging to a certain region in Germany, and thus exploring this ethnic identity can be different from their national identity. They may have considered national identity as a very broad categorization, while their ethnic, family heritage identity could be referred to as more differentiated and concrete, capturing those ethnic‐cultural aspects of their individual perceptions of Germany or a region in Germany that are actually present, tangible and passed on in their family. To better understand why national identity development was not promoted, it would have been helpful to know how majoritized students differentiate German national and ethnic identity and whether for example their regional identification or identification as East or West German could explain why ethnic, but not national, identity was not promoting global identity coherence, although both identities are to some extent overlapping. It would further have been valuable to explore whether minoritized and majoritized students thought of national identity in a stereotypical and exclusive, ancestry‐based or rather a more inclusive, residence‐based way (Ditlmann and Kopf‐Beck 2019).

The second research question (RQ2) investigated how adolescents' ethnic and national identity relate to global identity coherence. Unexpectedly, national identity was unrelated to global identity coherence. Perhaps discussions of national identity needed to be more in‐depth to help students understand how it may relate to their overall sense of self, especially because national identity is rarely discussed in Germany, and even less so in a pluralistic way. Or, perhaps an integration of national identity into adolescents' overall identity requires more time.

In contrast, in both the intervention and control group, minoritized and majoritized youth who gained more clarity of their ethnic identity reported less identity confusion, which does support the promotive links of ethnic identity resolution on more global identity coherence for adolescents (Safa et al. 2024). Moreover, when testing for result robustness, some of the alternative models (see Tables S2–S5) point to a trend that ethnic identity exploration may be challenging for the development of a coherent sense of self by being associated with more confusion and less synthesis for both minoritized and majoritized adolescents. Negative links of ethnic identity exploration and the positive links of resolution on identity coherence were found in other studies for both minoritized and majoritized college students (Syed and Juang 2014). Perhaps engaging in information seeking provokes processing new information, which may not be helpful and easy to navigate into but instead challenging for developing an overall sense of self (De Lise et al. 2024). When adolescents already gained more clarity about what an identity domain means for them, this can be supportive for developing a coherent identity.

Our study confirms that ethnic identity development mediates intervention effects on developing a coherent overall sense of self. Majoritized adolescents in the intervention (compared to those in the control), who were promoted in their ethnic identity resolution, showed less global identity confusion and more synthesis. This supports the promotive effect of the Identity Project intervention on adolescents global identity coherence (Ceccon, Moscardino et al. 2024; Umaña‐Taylor et al. 2018), at least for majoritized adolescents. On average, both minoritized and majoritized students' global identity coherence decreased over time. During adolescence and for the development of a coherent overall sense of self, identities other than ethnic and national identity can be meaningful, for example social identities like gender or sexual identity, or personal identities like educational identity (Benish‐Weisman et al. 2015; Crocetti et al. 2018). Perhaps adolescents engaged with other social identities at the same time period as the intervention took place. Reconsidering identity commitments or being uncertain about what those other identities mean for them may have inhibited a stronger overall sense of self. Perhaps for majoritized adolescents, in contrast to minoritized adolescents, coming to more clarity about their ethnic identity through the Identity Project was meaningful despite potentially engaging with other social identities, as they may not have thought much about their ethnic identity in relation to their overall sense of self before the intervention. For minoritized youth, their ethnic identity may already have been an important part of their daily lives and in social interactions, also in school.

Based on our findings, interventions that target ethnic and national identity exploration should necessarily also consider strategies to promote clarity after a period of information seeking and processing. This can be especially meaningful during adolescence when students engage with and navigate several different identity domains, which could be challenging for developing a coherent overall sense of self (Crocetti et al. 2024). For adolescents, discussions about what they learned and how their learnings help them to understand themselves better (Wantchekon et al. 2021), or opportunities for discussing what they found challenging after gaining more knowledge about ethnic and national groups (e.g., Germany's Nazi history, stereotypes about their ethnic group, or shared stories of discrimination and racism) and how they dealt with this information could be necessary and helpful if the process of resolution is guided in the protective environment of the classroom. Moreover, perhaps limited and weaker findings on global identity coherence could be related to the adaptation of the intervention from mid‐ to early‐adolescence (Bogaerts et al. 2021). Due to cognitive and psychosocial maturity, younger participants in both the group of minoritized and majoritized students may be less able to deeply reflect on their identities. They may need more guidance and time to process information and experiences made in the intervention, and how that is related to their own ethnic and national identities, to develop a clearer sense of self.

Our study has some limitations. We explored path difference testing and proceeded analysis with a partly constrained model, despite invariance of the fully unconstrained and the fully constrained model. Although this was based on theoretical and empirical evidence to assume differences and to prevent misspecification due to undetected, subtle differences between the groups, this procedure may carry the risk of a type I error. In both the fully and partly constrained model, ethnic identity resolution is associated with less global identity confusion. However, the intervention effects and indirect effects are supported with the partly constrained model and in the alternative models where minoritized and majoritized adolescents are separated, but not in the fully constrained model and those that consider the whole group.

Another limitation is that we did not investigate how global identity coherence contributes to adolescents' psychological and academic well‐being (see Safa et al. 2024). Future longitudinal studies could test how ethnic and national identity contribute to global identity coherence and, in turn, well‐being and academic engagement of minoritized and majoritized youth in immigration societies like Germany. Future intervention studies should examine how an inclusive and pluralist national identity can be best conveyed and promoted in school, especially in Germany, where national identification is linked to better academic achievement. Furthermore, how interventions can more strongly support adolescents in how they can successfully integrate both identities into a clear understanding of their own overall identity should be explored. More follow‐up data collections would have been helpful to examine whether the intervention was impactful for students ethnic and national identity development and their overall identity coherence in the longer term, also in terms of later reconsiderations of identity commitments. With more follow‐up data, it would further be interesting to investigate whether the German Identity Project contributes to the mixed findings of other European studies or supports the strict cascades of the theory of change underlying the intervention. Researchers could also track participation to test whether dosage is related to intervention effectiveness. Finally, future school intervention studies could include qualitative data to examine how minoritized and majoritized adolescents' understanding of a national identity can change through such an intervention, how ethnic and national identity are reflected and present in students' self‐identifications and whether their self‐identifications contain regional (e.g., Berliner), hyphenated (e.g., Vietnamese‐German), racialized (e.g., Black), or other overarching identities (e.g., European, Muslim, East‐German). With mixed‐methods studies and the collection of interview data, researchers could emphasize insights into the intersectionality of several identities and how identities other than national and ethnic identity were supportive or challenging for adolescents' global identity coherence.

## 10 Conclusion

The present study contributes to a better understanding of how ethnic and national identities of minoritized and majoritized adolescents can be promoted and how these identities can contribute to developing a coherent overall identity after participating in a school‐based identity intervention. We conclude that congruent to the initial main focus of the Identity Project intervention on ethnic(‐racial) identity, primarily students' ethnic identity but not their national identity was promoted. Our study also seems to support the *universality of the intervention* as it prompted both minoritized and majoritized adolescents to explore their ethnic identities. Furthermore, majoritized students who participated in the Identity Project were promoted in their ethnic identity resolution postintervention, which in turn was beneficial for their global identity coherence in terms of less confusion and more synthesis in a follow‐up timepoint. Minoritized students already had strong clarity about what their ethnic identity means to them at the baseline, and thus may have been limited in increasing their ethnic identity resolution. To support adolescents growing up in ethnic‐culturally diverse societies in finding out who they are, adolescents should be encouraged in school to engage with and more importantly come to more clarity with potential meaningful social identities, like their ethnic identity. More research is needed to examine the role of national identity for global identity coherence of youth in immigration societies and how a pluralist and inclusive understanding of national identity in Germany can effectively be promoted in school.

## 11 How Is the Article Community‐Based and Psychology‐Related?

Youth are part of several *communities*, for example, ethnic‐cultural communities, communities based on national residence but also school or classroom communities. The study goes beyond individualistic approaches: we highlight individual students as *members of social groups* and investigate how important certain social identities, namely ethnic and national identities, are for developing an overall sense of self in ethnic‐culturally diverse school contexts in Germany.

The study targets relevant *developmental psychological constructs*: ethnic and national identity development and global identity coherence, that all address identity development as a major developmental task during and beyond adolescence (Erikson 1968). We further emphasize an *equity perspective*, as we aim to contribute to educational equity for all students in German school contexts by understanding identity‐related factors that support psychosocial and academic adaptation (e.g., Umaña‐Taylor et al. 2014).

*Ethnic identities* can provide group memberships that transfer belongingness and provide guidance for youth' identity development. These identities can be meaningful for all (majoritized and minoritized) adolescents, for example, through shared traditions. These identities can further be protective factors and empowering especially for ethnic‐culturally minoritized students in Germany, for example, through shared coping strategies for experiencing racism, or re‐defining the perception of one's own group memberships.

*National identities* can provide a sense of belonging and, theoretically, provide a unifying, overarching identity for people with diverse ethnic identities growing up in a migration society like Germany. A differentiated, pluralistic understanding of their national (German) identity is also relevant for ethnic‐culturally majoritized students. Due to rising right‐wing‐populism hijacking debates about national identity content, values and belonging in an exclusive, racist, white supremacist way, an alternative meaning of their national identity must be offered.

The study concludes with *guidance for researchers and educators* when addressing and engaging with students ethnic and national identities in school to (1) offer opportunities for exploring and coming to more clarity regarding both identities for all students, and (2) reflect on the complexity of both identities in pluralistic societies with a rich migration history like Germany.

## Author contributions

Sharleen Pevec‐Zimmer was involved in the study conception and design, the adaptation of the Identity Project for German school contexts, moderated intervention sessions and collected intervention and control group data in Berlin, performed the statistical analyses, participated in the interpretation of the data, and drafted and revised the manuscript; Tuğçe Aral was involved in the study conception, supervised the statistical analyses, participated in the analyses interpretation, and revised and approved the manuscript; Linda P. Juang was involved in the study conception and design, the adaptation of the Identity Project for German school contexts, collected data of the German Identity Project in Berlin and revised and approved the manuscript; Maja K. Schachner was involved in the study conception and design, in the adaptation of the Identity Project for German school contexts, moderated intervention sessions in Berlin, collected data of the German Identity Project in Saxony‐Anhalt and revised and approved the manuscript.

## Consent

Written informed parental or legal guardian consent was obtained. All students participated in the Identity Project intervention as this was a regular school requirement. Students without consent did not participate in the survey.

## Conflicts of Interest

The authors declare no conflicts of interest.

## Supporting information

Supplemental Materials 17.

## Data Availability

The data that support the findings of this study are available on request from the corresponding author. The data are not publicly available due to privacy or ethical restrictions.

## Endnotes

1 We chose the terms ‘minoritized’ and ‘majoritized’ groups, adolescents or students, to highlight that certain ethnic‐cultural groups were and still are marginalized through social, political, and historical structures of power (Delgado and Stefancic 2012). This is important, because the negative implications of being minoritized or marginalized can persist even if those groups become the numerical majority.

2 Studies in the US use the terms ethnic, racial, and ethnic‐racial identity (Umaña‐Taylor et al. 2014). In Europe, racial identity is not a common term (see Juang et al. 2022). Hence, we refer to ethnic(‐racial) identity when referring to US‐studies, and ethnic identity when referring to studies in Europe.

3 Identity targets the developmental processes, while identification concerns self‐labeling at a certain timepoint (Umaña‐Taylor et al. 2014). The self‐categorization (e.g., strongly identifying with the family ethnic culture) emerged within the research field of acculturation and does not per se allow drawing conclusions of the processes that the person undertook to explore or commit to this identity, yet is helpful to understand the acceptance and rejection of these identities for individuals. As studies on ethnic and national identity development are still rare in Germany, we wish to contribute to this study field and as an addition to the strong research conducted on acculturation in Germany.

4 The Identity Project is a universal intervention as it was developed for a broad population including minoritized and majoritized adolescents (Umaña‐Taylor and Douglass 2017; Umaña‐Taylor et al. 2018). Therefore, the strategies, activities and input in the intervention to promote adolescents' in their ethnic‐racial exploration, and subsequently their resolution, can be helpful for adolescents of diverse ethnic‐racial identities (Umaña‐Taylor and Douglass 2017).

5 For transparency, the analysis results of the fully constrained model are shown in the Supplemental Figure S15. Despite a significant *χ* ^2^ difference, invariance of the fully and partly constrained model holds (*χ* ^2^Δ(1) = 4.099, *p* = 0.043, ΔCFI = 0.003, ΔRMSEA = 0.001, ΔSRMR = 0.0000). Regarding the results, only the significance of the b3 path for both groups and regardless of the intervention is supported. As the intervention effect on ethnic identity exploration is supported by Hölscher et al. (2024), who used a subsample from the present study and included minoritized status as a covariate, and Schachner et al. (2024), we assume the intervention effect is masked when all paths are constrained. Not considering differences for minoritized versus majoritized adolescents in the fully constrained model and the supplemental analyses with the whole group, see Figures S4, S5, and S15, may thus be too restrictive and misspecified, so that intervention effects are suppressed (Hsiao and Lai 2018).

6 All four mediators were positively predicted by the previous (T1) same‐scale measurement for both groups with coefficients ranging from *β* = 0.165 to 0.553. For minoritized students, T1 ethnic identity exploration positively predicted T2 ethnic identity resolution (*β* = 0.131, *p* = 0.032) and T1 national identity exploration positively predicted T2 national identity resolution (*β* = 0.145, *p* = 0.010). For majoritized but not minoritized adolescents, T1 national identity exploration positively predicted T2 ethnic identity exploration (*β* = 0.216, *p* = 0.006), T1 national identity resolution positively predicted T2 ethnic identity resolution (*β* = 0.213, *p* = 0.024) and T1 ethnic identity exploration positively predicted T2 national identity exploration (*β* = 0.200, *p* = 0.003).

7 To mimic the theory of change of the original US Identity Project study (Umaña‐Taylor et al. 2018), this alternate analysis was modeled with the whole group and with limited timepoints of our T1–T3 data. As testing for the strict cascades is not possible, we instead explored whether the intervention promoted T2 exploration, which subsequently promoted T2 resolution, which then promoted T3 global identity coherence.
