# Supplementary material for: Promoting Social Identities as Resources—The Role of Ethnic and National Identity Development for Adolescents' Global Identity Coherence in Ethnic‐Culturally Diverse Schools in Germany
Source: J Community Psychol. 2025 Nov 14;53(8):e70059. doi: 10.1002/jcop.70059 (PMC12616773; doi:10.1002/jcop.70059)
Supplement: Supplementary file 1 — Supplemental Materials 17. [file JCOP-53-0-s001.docx]

**Supplemental Materials**

|  | **Supplemental Table S1:** Ethnic-cultural heritages and sample proportions of mono- and bi-/multicultural self-identifications | p. 2 |
| --- | --- | --- |
|  | **Supplemental Figure S2:** Path coefficients and mediation effects for minoritized vs. majoritized students without FIML data imputation | p. 3 |
| **Supplemental Figure S3:** Path coefficients and mediation effects for minoritized vs. majoritized students including age and region as covariates | | p. 4 |
| **Supplemental Figure S4:** Path coefficients and mediation effects for the whole sample | | p. 5 |
|  | **Supplemental Figure S5:** Path coefficients and mediation effects for minoritized vs. majoritized students, modelled to mimic the theory of change with limited data timepoints T1-T3 for the whole sample | p. 6 |
|  |  |  |
|  | **Supplemental Table S6:** Measurement invariance of national identity development (exploration and resolution) by ethnic-culturally minoritized/majoritized status | p. 7 |
|  | **Supplemental Table S7:** Measurement invariance of ethnic identity development (exploration and resolution) by ethnic-culturally minoritized/majoritized status | p. 7 |
|  | **Supplemental Table S8:** Measurement invariance of global identity development (confusion and synthesis) by ethnic-culturally minoritized/majoritized status | p. 7 |
|  | **Supplemental Table S9:**  Measurement invariance of national identity exploration across time | p. 8 |
|  | **Supplemental Table S10:** Measurement invariance of national identity resolution across time | p. 8 |
|  | **Supplemental Table S11:** Measurement invariance of ethnic identity exploration across time | p. 8 |
|  | **Supplemental Table S12:** Measurement invariance of ethnic identity resolution across time | p. 8 |
|  | **Supplemental Table S13:** Measurement invariance of global identity confusion across time | p. 9 |
|  | **Supplemental Table S14:** Measurement invariance of global identity synthesis across time | p. 9 |
|  | **Supplemental Figure S15:** Path coefficients and mediation effects for minoritized vs. majoritized students with all paths constrained to be equal | p. 10 |
|  |  |  |

Supplemental Table S1

*Ethnic-cultural heritages and sample proportions of mono- and bi-/multicultural self-identifications*

| \|  \|  \| \| --- \| --- \| \| **Mono-, bi- or multicultural self-identification** \| **Percentage of sample (%)** \| \| Monocultural German self-identification \| 27.2% (*N* = 158) \| \| Bi- or multicultural self-identification \| 38.7 (*N* = 225) \| \| Missing/no information provided \| 34.1% (*N* = 198) \| \|  \|  \| \| **Heritage cultural groups chosen as self-identification categories in the sample, per region** \| ***N* present in the sample** \| \| North-Western Europe (e.g. Germany, France) \| 292 \| \| Southern Europe (e.g. Greece, Italy) \| 10 \| \| Eastern Europe, Russia and Balkan (e.g. Poland, Russia, Bulgaria, Serbia) \| 76 \| \| Middle East and North Africa (e.g. Turkey, Syria, Marocco) \| 127 \| \| Subsahara (e.g. Nigeria, Somalia) \| 16 \| \| South-East-Asia (e.g. Vietnam, India) \| <10 \| \| North and South America (e.g. USA, Dominican Republic) \| <10 \| \| Australia and New Zealand (e.g. Australia) \| <10 \| |
| --- | --- | --- | --- | --- | --- | --- | --- | --- | --- | --- | --- | --- | --- | --- | --- | --- | --- | --- | --- | --- | --- | --- | --- | --- | --- | --- | --- | --- | --- | --- |
| *Note. Information of adolescents‘ self-identification come from two self-identification items in the questionnaire: an open-ended questions regarding their heritage culture „What would you say is your heritage culture or are your heritage cultures? As you already know, this term means the family background of a person, for example where the parents or grandparents come from! It can also be related to a specific region, for example in Germany” and one multiple-choice-question with that captured the ethnic-cultural self-identification considering family heritage cultures and growing up in Germany “Some people see themselves as German, others as Turkish, and others as German-Turkish. How is this with you? How do you see yourself?“. The multiple choice options were related to the largest ethnic-cultural minorities in Germany: “German, Turkish, Turkish-German, Russian, German-Russian, Polish, German-Polish, Syrian, German-Syrian, Something else, that is* _______*.”. A total of 383 participants provided self-identification information. As participants could endorse identifications with more than one ethnic-cultural group, the number of reported identifications exceeds the number of participants that provided information. Additionally, as nonresponse of 198 participants resulted in missing data, such that the total number of responses is less than the full sample size of N = 581.* |

|  |  |
| --- | --- |

Supplemental Figure S2:

*Path coefficients and mediation effects for minoritized vs. majoritized students without FIML data imputation (model for sensitivity analysis)*

| 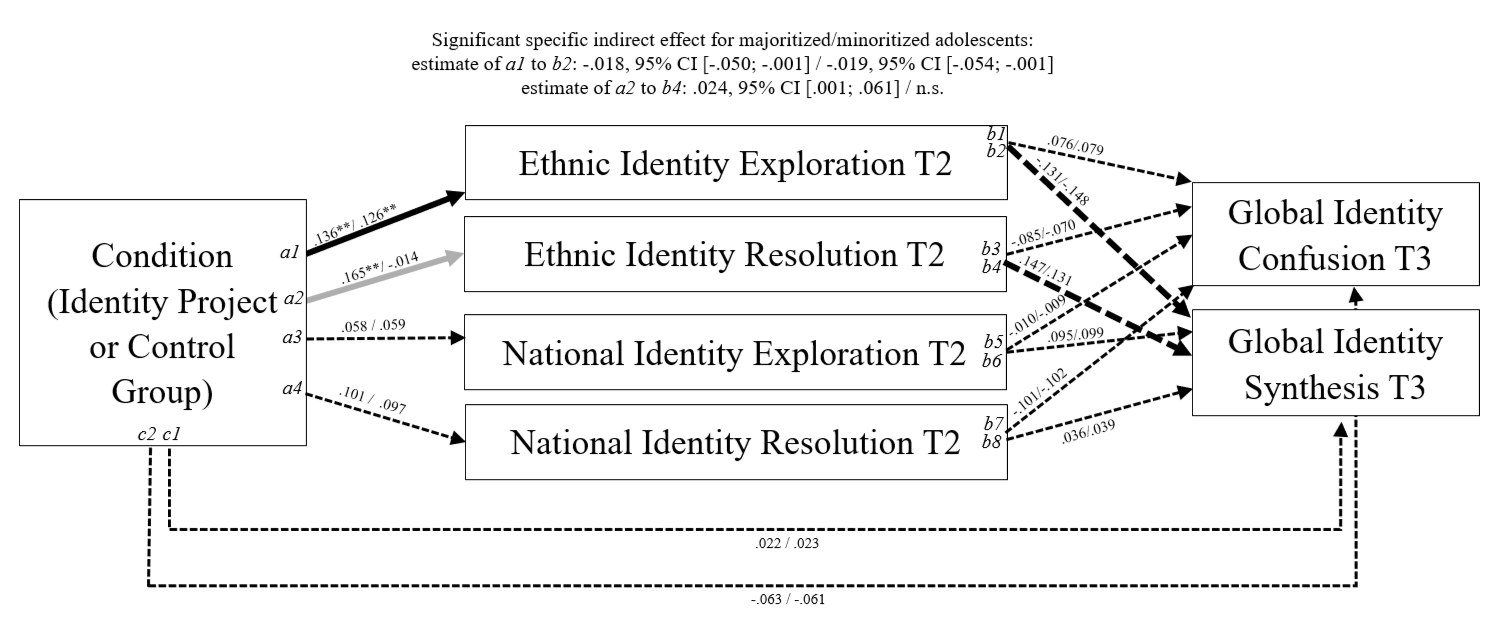 |
| --- |
| *Note.* *N* = 276 (minoritized adolescents *n* = 141, majoritized adolescents *n* = 135). Standardized path coefficients are reported for majoritized/minoritized adolescents, with estimates for majoritized adolescents before and for minoritized adolescents after the slash. Dashed line indicates non-significant path for both groups. Light grey line indicates significant path for one of the groups. Solid black line indicates significant path for both groups. Bold lines indicate significant indirect effects. Dashed bold lines indicate a significant indirect effect despite insignificant a path and/or b path. The results derive from the conceptual model shown in figure 1, including covariates and correlations. **p* < .05, ** *p* < .01, *** *p* < .001. Model fit = *χ^2^/df* (97) = 176.744, *p* = .000, CFI = .913, TLI = .876, RMSEA = .077, 90% CI = [.059, .095], SRMR = .065. |

| Supplemental Figure S3 |
| --- |
| *Path coefficients and mediation effects for minoritized vs. majoritized students including cohort, region and age as covariates (model for sensitivity analysis)* |
| 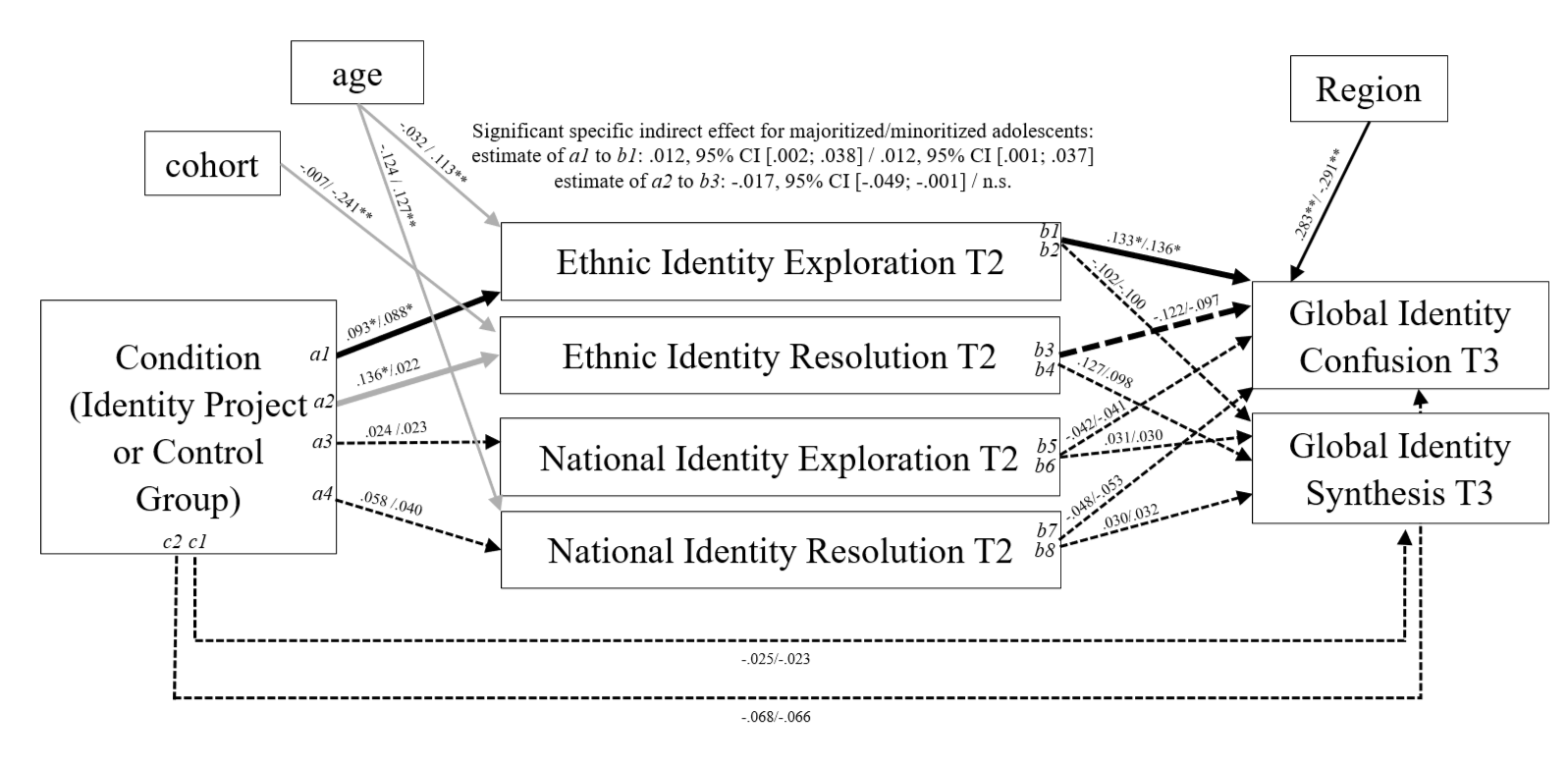 |
| *Note.* *N* = 581 (minoritized adolescents *n* = 331, majoritized adolescents *n* = 250). Standardized path coefficients are reported for majoritized/minoritized adolescents, with estimates for majoritized adolescents before and for minoritized adolescents after the slash. Dashed line indicates non-significant path for both groups. Light grey line indicates significant path for one of the groups. Solid black line indicates significant path for both groups. Bold lines indicate significant indirect effects. Dashed bold lines indicate a significant indirect effect despite insignificant a path and/or b path. The results derive from the conceptual model shown in figure 1, including covariates and correlations, with the additional covariates of cohort, region and age. **p* < .05, ** *p* < .01, *** *p* < .001; cohort: 2018/19 = 1, 2019/20 = 2, 2021/22 = 3; age = 11-16 years; region: Saxony-Anhalt = 0, Berlin = 1. Model fit = *χ^2^/df* (145) = 307.706, *p* = .000, CFI = .895, TLI = .831, RMSEA = .064, 90% CI = [.054, .074], SRMR = .078. |
| Supplemental Figure S4 |
| *Path coefficients and mediation effects for the whole sample (model for sensitivity analysis)* |
| 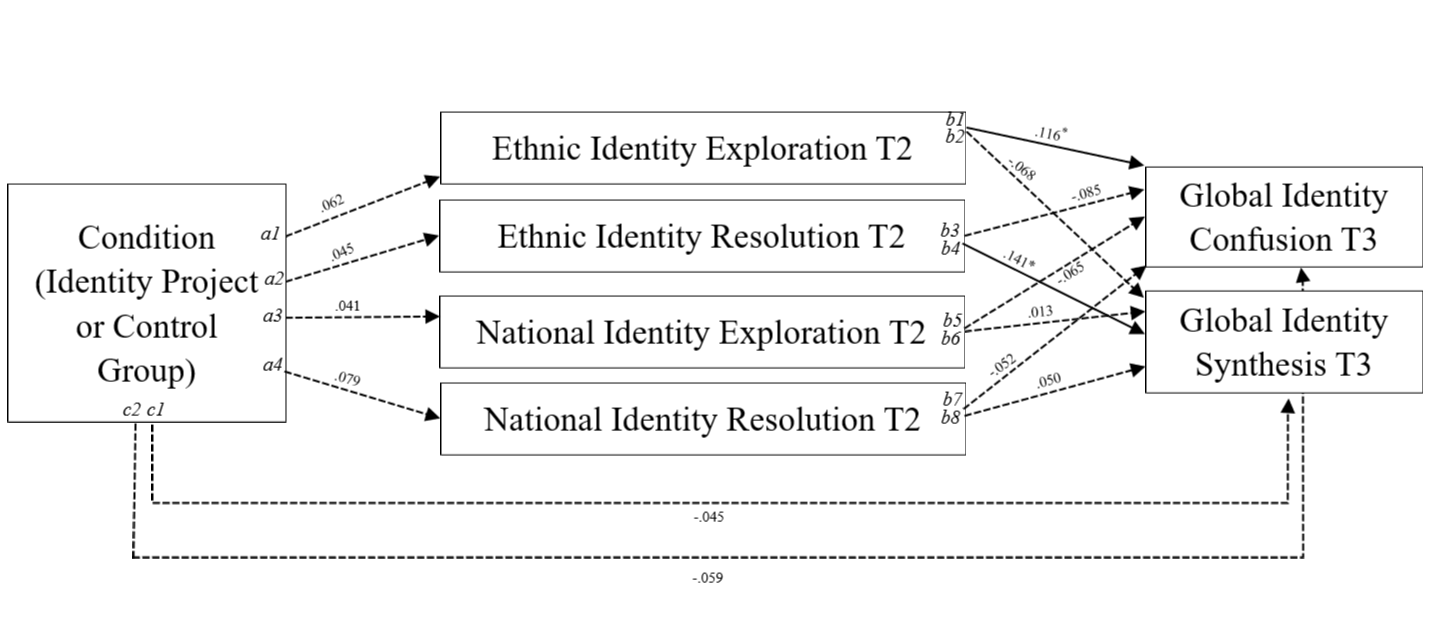 |
| *Note.* *N* = 581. Significant standardized path coefficients are reported. Dashed line indicates non-significant path. Solid black line indicates significant path. The model indicated no significant indirect effects. The results derive from the conceptual model shown in figure 1, including covariates and correlations. **p* < .05, ** *p* < .01, *** *p* < .001. Model fit = *χ^2^/df* (42) = 135.489, *p* = .000, CFI = .922, TLI = .872, RMSEA = .062, 90% CI = [.050, .074], SRMR = .045. |

| Supplemental Figure S5 |
| --- |
| *Path coefficients and mediation effects for minoritized vs. majoritized students, modelled to mimic the theory of change with limited data timepoints T1-T3 for the whole sample (model for sensitivity analysis)* |
| 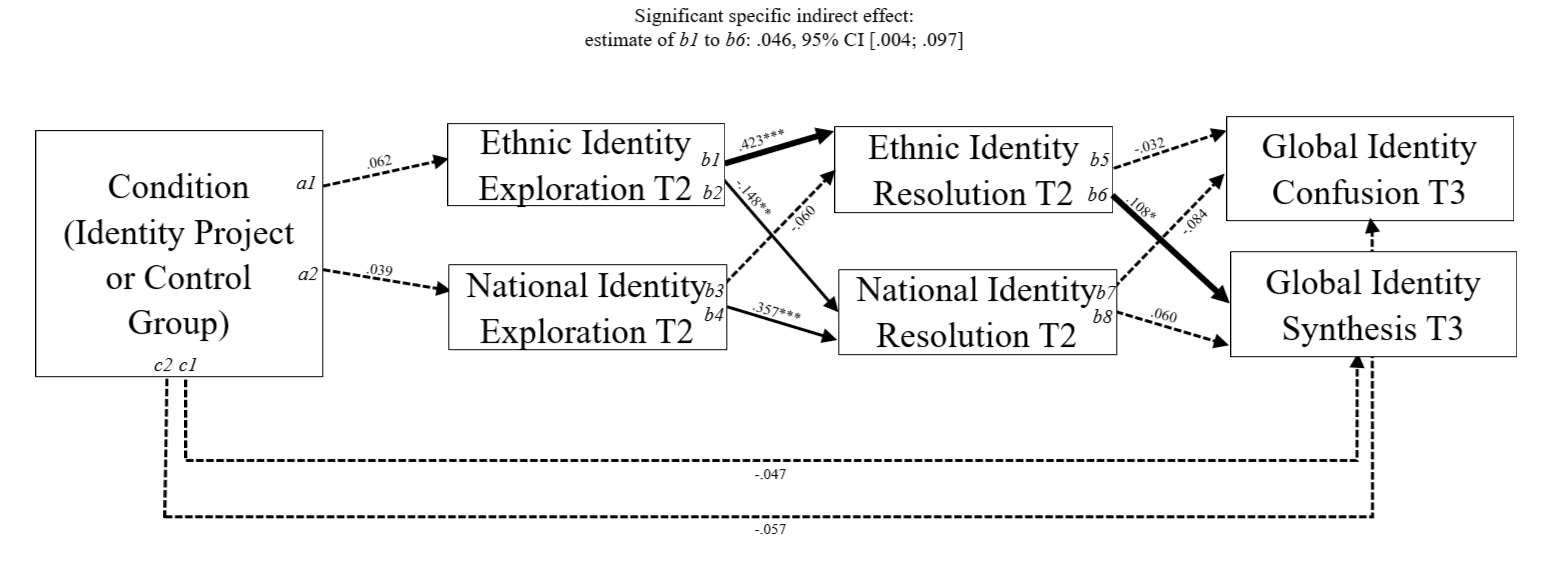 |
| *Note.* *N* = 581 (minoritized adolescents *n* = 331, majoritized adolescents *n* = 250). Standardized path coefficients are reported. Dashed line indicates non-significant path. Solid black line indicates significant path. Bold lines indicate significant indirect effects. The results derive from an adapted version of the conceptual model shown in figure 1, including covariates and correlations. The model was adapted to mimic the cascades of the theory of change with limited T1-T2-T3 data. **p* < .05, ** *p* < .01, *** *p* < .001. Model fit = *χ^2^/df* (48) = 143.684, *p* = .000, CFI = 920, TLI = .886, RMSEA = .059, 90% CI = [.048, .070], SRMR = .045. |

**Supplemental Table S6.** *Measurement invariance of national identity development (exploration and resolution) by ethnic-culturally minoritized/ majoritized status*

|  | *χ*^2^(df) | CFI | RMSEA [90% CI] | SRMR | ΔCFI | ΔRMSEA | ΔSRMR |
| --- | --- | --- | --- | --- | --- | --- | --- |
| Configural | 20.608 (16) | .995 | .034 [.000, .072] | .026 | – | – | – |
| Metric | 25.512 (20) | .994 | .034 [.000, .068] | .039 | -.001 | .000 | .013 |
| Scalar | 41.919 (24) | .982 | .055 [.025, .082] | .052 | -.012 | .021 | .013 |

*Note. N* = 490 (minoritized adolescents *n* = 276, majoritized adolescents *n* = 214).

**Supplemental Table S7.** *Measurement invariance of ethnic identity development (exploration and resolution) by ethnic-culturally minoritized/ majoritized status*

|  | *χ*^2^(df) | CFI | RMSEA [90% CI] | SRMR | ΔCFI | ΔRMSEA | ΔSRMR |
| --- | --- | --- | --- | --- | --- | --- | --- |
| Configural | 34.736 (16) | .985 | .069 [.037, .101] | .026 | – | – | – |
| Metric | 44.010 (20) | .981 | .070 [.042, .098] | .048 | -.004 | .001 | .022 |
| Scalar | 52.804 (24) | .977 | .070 [.044, .096] | .045 | -.004 | .000 | -.003 |

*Note. N* = 487 (minoritized adolescents *n* = 274, majoritized adolescents *n* = 213).

**Supplemental Table S8.** *Measurement invariance of global identity development (confusion and synthesis) by ethnic-culturally minoritized/ majoritized status*

|  | *χ*^2^(df) | CFI | RMSEA [90% CI] | SRMR | ΔCFI | ΔRMSEA | ΔSRMR |
| --- | --- | --- | --- | --- | --- | --- | --- |
| Configural | 86.015 (38) | .922 | .071 [.051, .091] | .054 | – | – | – |
| Metric | 92.378 (44) | .921 | .067 [.047, .086] | .061 | -.001 | -.004 | .007 |
| Scalar | 102.776 (50) | .914 | .065 [.047, .083] | .065 | -.007 | -.002 | .004 |

*Note. N* = 496 (minoritized adolescents *n* = 281, majoritized adolescents *n* = 215).

**Supplemental Table S9.** *Measurement invariance of national identity exploration across time*

|  | *χ*^2^(df) | CFI/TLI | RMSEA [90% CI] | SRMR | ΔCFI | ΔRMSEA | ΔSRMR |
| --- | --- | --- | --- | --- | --- | --- | --- |
| Configural | 31.561 (8) | .972/.948 | .072 [.047, .100] | .038 | – | – | – |
| Metric | 37.021 (10) | .968/.952 | .069 [.046, .094] | .046 | -.004 | -.003 | .008 |
| Scalar | 37.027 (11) | .969/.958 | .065 [.043, .088] | .046 | .001 | -.004 | .000 |

*Note.* *N* = 564.

**Supplemental Table S10.** *Measurement invariance of national identity resolution across time*

|  | *χ*^2^(*df*) | CFI/TLI | RMSEA [90% CI] | SRMR | ΔCFI | ΔRMSEA | ΔSRMR |
| --- | --- | --- | --- | --- | --- | --- | --- |
| Configural | 22.151 (8) | .990/.981 | .056 [.029, .085] | .018 | – | – | – |
| Metric | 27.787 (10) | .987/.981 | .056 [.032, .082] | .036 | -.003 | .000 | .018 |
| Scalar | 30.753 (11) | .986/.981 | .057 [.033, .081] | .038 | -.001 | .001 | .002 |

*Note.* *N* = 561.

**Supplemental Table S11.** *Measurement invariance of ethnic identity exploration across time*

|  | *χ*^2^(*df*) | CFI/TLI | RMSEA [90% CI] | SRMR | ΔCFI | ΔRMSEA | ΔSRMR |
| --- | --- | --- | --- | --- | --- | --- | --- |
| Configural | 22.881 (8) | .990/.982 | .058 [.031, .086] | .025 | – | – | – |
| Metric | 23.153 (10) | .991/.987 | .048 [.022, .074] | .025 | .001 | -.010 | .000 |
| Scalar | 23.159 (11) | .992/.989 | .044 [.018, .070] | .025 | .001 | -.004 | .000 |

*Note.* *N* = 562.

**Supplemental Table S12.** *Measurement invariance of ethnic identity resolution across time*

|  | *χ*^2^(*df*) | CFI/TLI | RMSEA [90% CI] | SRMR | ΔCFI | ΔRMSEA | ΔSRMR |
| --- | --- | --- | --- | --- | --- | --- | --- |
| Configural | 23.757 (8) | .990/.982 | .059 [.033, .087] | .024 | – | – | – |
| Metric | 26.393 (10) | .990/.985 | .054 [.029, .080] | .034 | .000 | -.005 | .010 |
| Scalar | 28.442 (11) | .989/.985 | .053 [.029, .077] | .036 | -.001 | -.001 | .002 |

*Note.* *N* = 563.

**Supplemental Table S13.** *Measurement invariance of global identity confusion across time*

|  | *χ*^2^(*df*) | CFI/TLI | RMSEA [90% CI] | SRMR | ΔCFI | ΔRMSEA | ΔSRMR |
| --- | --- | --- | --- | --- | --- | --- | --- |
| Configural | 147.056 (24) | .871/.808 | .094 [.080, .109] | .061 | – | – | – |
| Metric | 148.652 (28) | .873/.837 | .086 [.073, .100] | .062 | .002 | -.008 | .001 |
| Scalar | 153.973 (29) | .868/.836 | .087 [.074, .100] | .063 | -.005 | .001 | .001 |

*Note.* *N* = 578.

**Supplemental Table S14.** *Measurement invariance of global identity synthesis across time*

|  | *χ*^2^(*df*) | CFI/TLI | RMSEA [90% CI] | SRMR | ΔCFI | ΔRMSEA | ΔSRMR |
| --- | --- | --- | --- | --- | --- | --- | --- |
| Configural | 303.794 (87) | .887/.863 | .066 [.058, .074] | .064 | – | – | – |
| Metric | 309.773 (95) | .888/.876 | .063 [.055, .070] | .067 | .001 | -.003 | .003 |
| Scalar | 362.502 (98) | .872/.852 | .068 [.061, .076] | .080 | -.016 | .005 | .013 |

*Note.* *N* = 578.

| Supplemental Figure S15 |
| --- |
| *Path coefficients and mediation effects for minoritized vs. majoritized students with all paths constrained to be equal* |
| 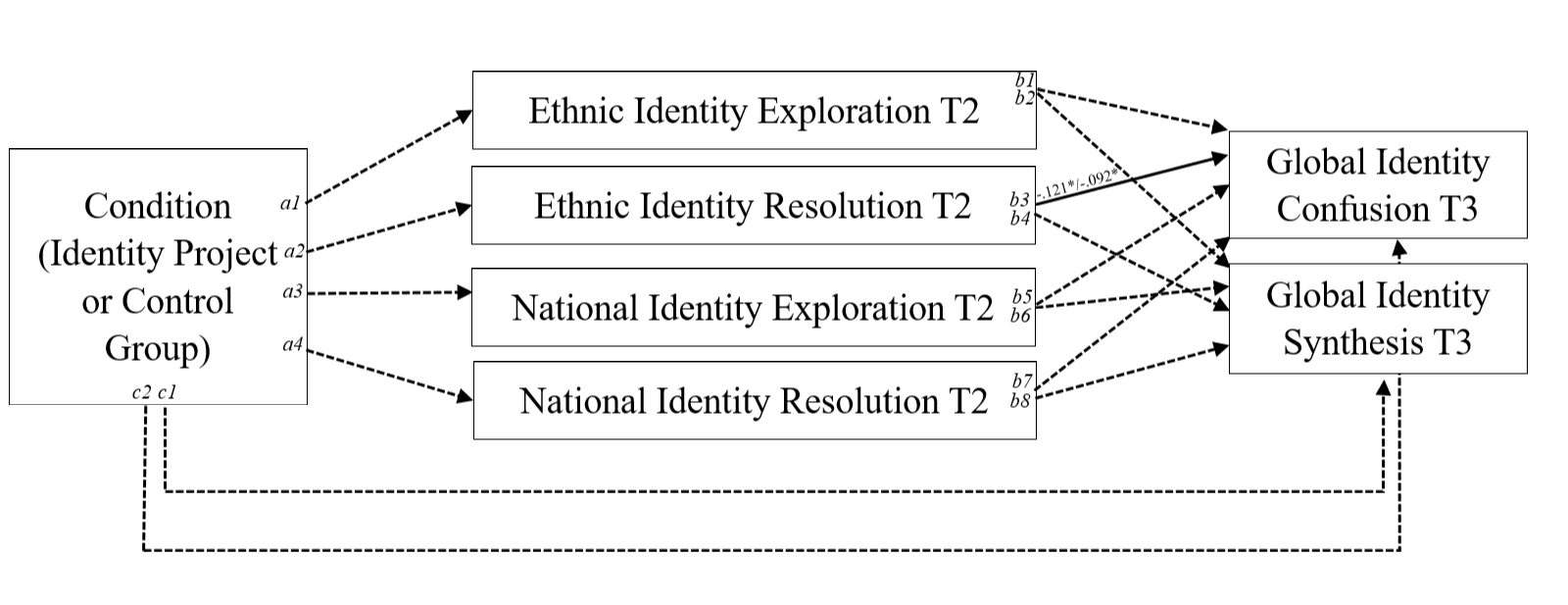 |
| *Note. N =* 581 (minoritized adolescents *n* = 331, majoritized adolescents *n* = 250). Standardized path coefficients are reported for majoritized/minoritized adolescents, with estimates for majoritized adolescents before and for minoritized adolescents after the slash. Dashed line indicates non-significant path for both groups. Solid black line indicates significant path for both groups. The model indicated no significant indirect effects. The results derive from the conceptual model shown in figure 1, including covariates and correlations. **p* < .05, ** *p* < .01, *** *p* < .001. Model fit = *χ^2^/df* (98) = 184.417, *p* = .000, CFI = .926, TLI = .896, RMSEA = .055, 90% CI = [.043, .067], SRMR = .054. |
